# Genetic Control of Tissue Remodeling by a Non‐Coding SNP in *ITGA8* Explains Carotenoid‐Based Color Polymorphism in Marine Mollusks

**DOI:** 10.1002/advs.202520527

**Published:** 2026-01-28

**Authors:** Xiaohui Wei, Wenzhu Peng, Zekun Huang, Bingye Yang, Yiyu Wu, Minzhen Han, Yi Wang, Bingling Peng, Guosheng Hu, Jiawei Hong, Guijia Liu, Linwei Nie, Yang Gan, Miaoqin Huang, Xuan Luo, Weiwei You, Wen Liu, Caihuan Ke

**Affiliations:** ^1^ State Key Laboratory of Mariculture Breeding College of Ocean and Earth Sciences Xiamen University Xiamen China; ^2^ Institutes of Brain Science State Key Laboratory of Medical Neurobiology and MOE Frontiers Center for Brain Science Fudan University Shanghai China; ^3^ Fujian Ocean Innovation Center Xiamen China; ^4^ College of the Environmental and Ecology Xiamen University Xiamen China; ^5^ Xiamen Key Laboratory of Marine Medicinal Natural Products Resources Xiamen Medical College Xiamen China; ^6^ State Key Laboratory of Cellular Stress Biology School of Pharmaceutical Sciences Faculty of Medicine and Life Sciences Xiamen University Xiamen China; ^7^ State Key Laboratory of Marine Environmental Science College of Ocean and Earth Sciences Xiamen University Xiamen China

**Keywords:** carotenoid‐based coloration, ITGA8, marine mollusk, non‐coding SNP, tissue remodeling

## Abstract

Biological color polymorphism represents a widespread form of phenotypic diversity in nature; however, the multi‐scale regulatory mechanisms of carotenoid‐based coloration, from genetic control to cell states and tissue architecture, remain poorly understood. Here, using the orange‐muscle giant abalone (*Haliotis gigantea*) as a model, we demonstrate that a non‐coding single nucleotide polymorphism (SNP) in the integrin subunit alpha 8 (*ITGA8*) gene induces alternative splicing, thereby promoting carotenoid‐based coloration through the regulation of tissue remodeling processes, including cell state transitions and reconstruction of the physical microenvironment. Through chromosome‐level genome assembly and genome‐wide association studies (GWAS), we identified a SNP that disrupts the interaction between *ITGA8* pre‐mRNA and the splicing factor ILF2, leading to altered *ITGA8* splicing. Multi‐omics analyses and transmission electron microscopy reveal that these splicing changes promote carotenoid accumulation in abalone muscle by weakening cell adhesion and enlarging intercellular spaces. Functional validation supports the role of *ITGA8* in regulating cell adhesion and carotenoid uptake. Our study uncovers a cross‐scale mechanism by which genetic variation drives macroscopic phenotypic diversity in carotenoid‐based color polymorphism through the regulation of cell state transitions and tissue remodeling. These findings provide novel insights into the genotype‐phenotype relationship and advance our understanding of color diversity in multicellular organisms.

## Graphical abstract

In this study, the orange‐muscle giant abalone (*Haliotis gigantea*) is used as a model to identify a non‐coding SNP that disrupts the interaction between *ITGA8* pre‐mRNA and the splicing factor ILF2, leading to altered *ITGA8* splicing. These splicing changes promote carotenoid accumulation in abalone muscle through the regulation of tissue remodeling processes, including weakened cell adhesion and expanded intercellular spaces.

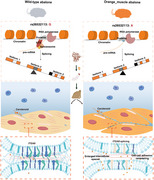

## 1 Introduction

Biological color polymorphism is a widespread form of phenotypic diversity in nature, and its complex formation mechanisms offer unique insights into the genetic, developmental, morphological, and evolutionary processes of organisms [1]. As a common phenotypic trait, the emergence of color polymorphism typically involves the coordinated regulation across chemical, biological, and physical dimensions. At the chemical level, it primarily concerns the absorption, biosynthesis, and metabolism of pigment molecules; at the biological level, it encompasses genetic variation, the reconfiguration of gene regulatory networks, and spatiotemporally specific developmental programs; at the physical level, it is closely related to the microstructural properties of cells and tissues. This multilayered regulatory network not only reflects the complexity of morphogenesis, but also exerts profound influences on metabolic regulation, physiological functions, and environmental adaptability [2]. Pigment deposition, nanostructural characteristics, and their interactions are major drivers of color polymorphism in organisms [3]; even pigment‐based coloration will be modulated by intrinsic physical structures within the body [4]. From birds with glossy red plumages [5] to ultra‐black peacock spiders [6], stick insects [7], butterflies [8], and flowers with cone‐shaped epidermal cells to generate more colorful petals [9], many species in nature employ internal structures to enhance the effects of pigments. Despite these examples, the ways in which internal structures influence pigment‐mediated color polymorphism, and how multiscale coordination among genetic regulation, cellular states, and tissue architecture is achieved, are poorly characterized.

Yellow, orange, and red colors are particularly common in birds, fish, crustaceans, and mollusks, primarily due to the deposition of carotenoids in their bodies. β‐carotene and zeaxanthin are two common carotenoids used for animal pigmentation, among which β‐carotene can be converted into vitamin A, while zeaxanthin possesses physiological functions in delaying and inhibiting various chronic diseases [10, 11, 12]. Previous studies on carotenoid‐based color polymorphism have primarily focused on the genetic regulatory mechanisms within key metabolic and transport pathways [13, 14, 15]. Because animals lack endogenous pathways for carotenoid synthesis, they must obtain these pigments from dietary sources or symbiotic organisms [16]. Ingested carotenoids are generally absorbed by intestinal cells through simple diffusion or specific transport proteins, subsequently incorporated into lipoproteins, and distributed to various tissues via the circulatory system [17, 18]. Lipoproteins are then deposited in target tissues through receptor‐mediated uptake, vesicular trafficking, and regulation of tissue permeability [19, 20]. Multicellular tissue homeostasis relies on coordinated cell–cell interactions, a structurally intact ECM network, and dynamic signaling pathways, all essential for preserving tissue architecture and barrier function. Disruptions such as diminished cell adhesion, compromised intercellular junctions, disorganization of the glycocalyx, and ECM remodeling can lead to widened intercellular spaces. These structural alterations compromise tissue integrity and consequently modulate the transcellular transport of exogenous substances, including carotenoids [21, 22]. Notably, differences in cell adhesion among multicellular tissues can reorganize ECM components and drive large‐scale tissue remodeling, ultimately leading to distinct morphological phenotypes [23, 24, 25]. Nevertheless, the mechanisms by which genetic variation regulates cellular states and tissue architecture, shapes carotenoid deposition, and contributes to pigment‐mediated color polymorphism remain poorly understood.

The mollusk, a simple marine invertebrate, has a distinctive open circulatory system (Figure S1), where hemolymph directly fills lacunar tissue spaces due to the absence of an endothelial lining. Changes in internal tissue organization may significantly impact the exchange of exogenous chemicals, including carotenoids, within tissues [26]. Giant abalone (*Haliotis gigantea*) has produced breeds with fixed expressions of distinct carotenoid‐based orange color phenotypes, which are inherited as a recessive trait [27]. Elucidating its formation mechanism provides an ideal model for uncovering how genes regulate tissue organization to control carotenoid deposition. In this study, by revealing the unique process of carotenoid accumulation in orange‐muscle abalone, we aim to understand how genetic factors regulate cellular states and tissue structures in animals, thereby providing comprehensive insight into the multiscale mechanisms underlying carotenoid‐based coloration. We uncovered a novel mechanism of carotenoid accumulation in animals. Specifically, we demonstrated a novel mechanism by which a non‐coding single‐nucleotide polymorphism (SNP) modulates the alternative splicing of *ITGA8*, leading to weakened cell adhesion and expanded intercellular spaces, thereby contributing to carotenoid coloration polymorphism in mollusks.

## 2 Results

### 2.1 Orange‐Muscle Abalone has a Higher Carotenoid Content

**FIGURE 1:**
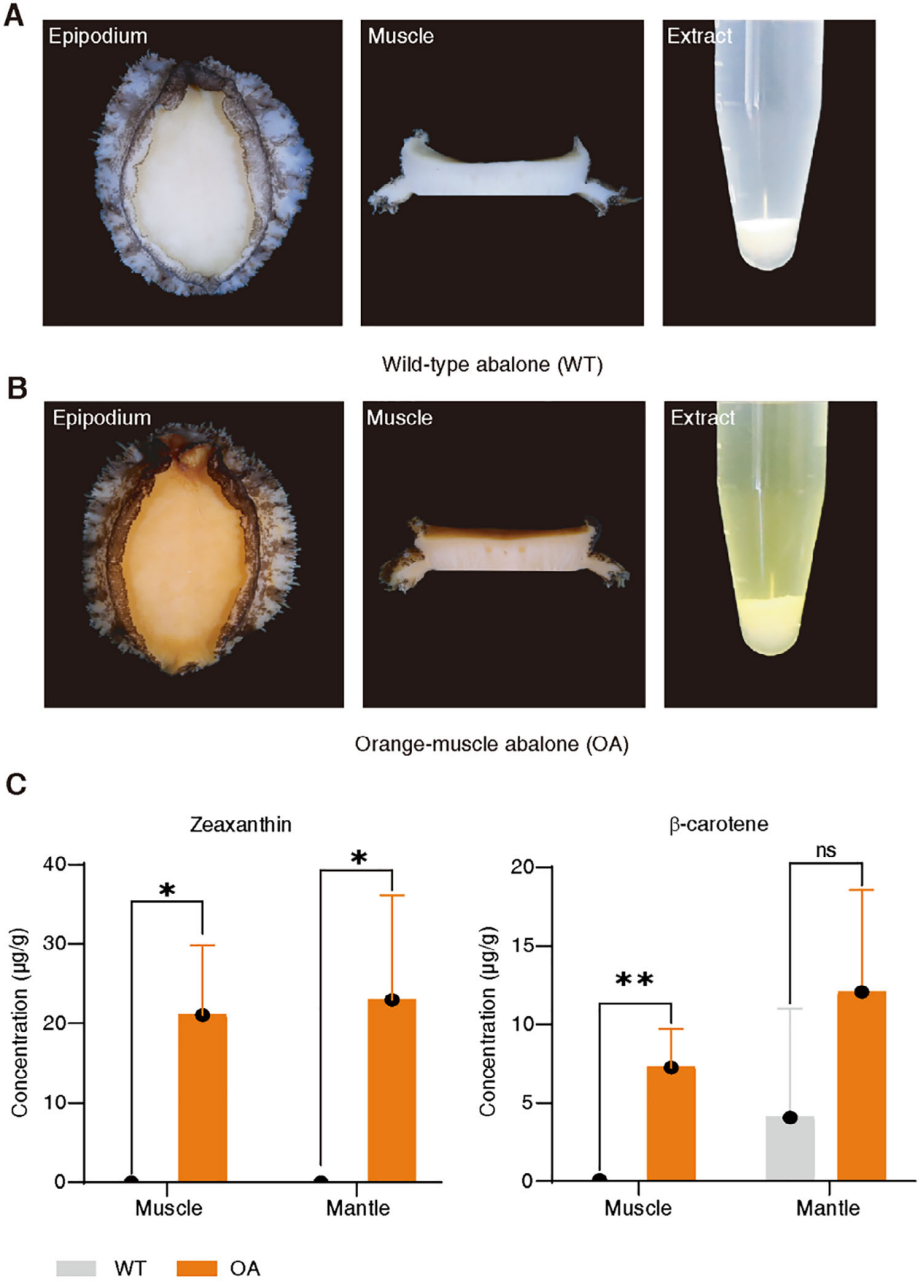
Carotenoid concentrations of wild‐type and orange‐muscle abalone. (A) and (B) Representative images of wild‐type abalone (WT) (A) and orange‐muscle abalone (OA) (B). Epipodium: View of the epipodium of the abalone; Muscle: Transverse section of the abalone muscle; Extract: Ethyl acetate extract from the abalone muscle. (C) Zeaxanthin (left) and β‐carotene (right) concentrations in the muscles and mantles of typical wild‐type and orange‐muscle abalone. The carotenoid concentrations were measured by normal‐phase HPLC. Data are expressed as the mean ± SD (n = 3). Statistical analyses: Student's *t*‐test. ^*^ *P* < 0.05; ^**^ *P* < 0.01; NS: Not significant.

The orange recessive abalone is characterized by orange muscle color that appears to have higher carotenoid pigmentation than typical wild‐type abalone (Figure 1A,B). To better understand the carotenoid composition of the orange‐muscle abalone, we used high‐performance liquid chromatography (HPLC) to identify and quantify carotenoids in the muscle and mantle, which are important sites of carotenoid accumulation in abalone. Two carotenoids, zeaxanthin and β‐carotene, were detected in the abalone, which is consistent with previous studies (Figure S2) [28]. The muscle of the orange‐muscle abalone showed significantly higher zeaxanthin content (21.04 ± 8.74 µg/g) compared to wild‐type abalone (*P* < 0.05). The β‐carotene content (7.17 ± 2.48 µg/g) was also significantly increased (*P* < 0.01). Moreover, the average zeaxanthin content in the mantle of the orange‐muscle abalone was significantly higher than that of wild‐type abalone (*P* < 0.05) (Figure 1C). Compared to wild‐type abalone, the carotenoid levels, especially the zeaxanthin content, were significantly increased in the tissues of the orange‐muscle abalone.

### 2.2 Genome Assembly of *H. Gigantea*

**FIGURE 2:**
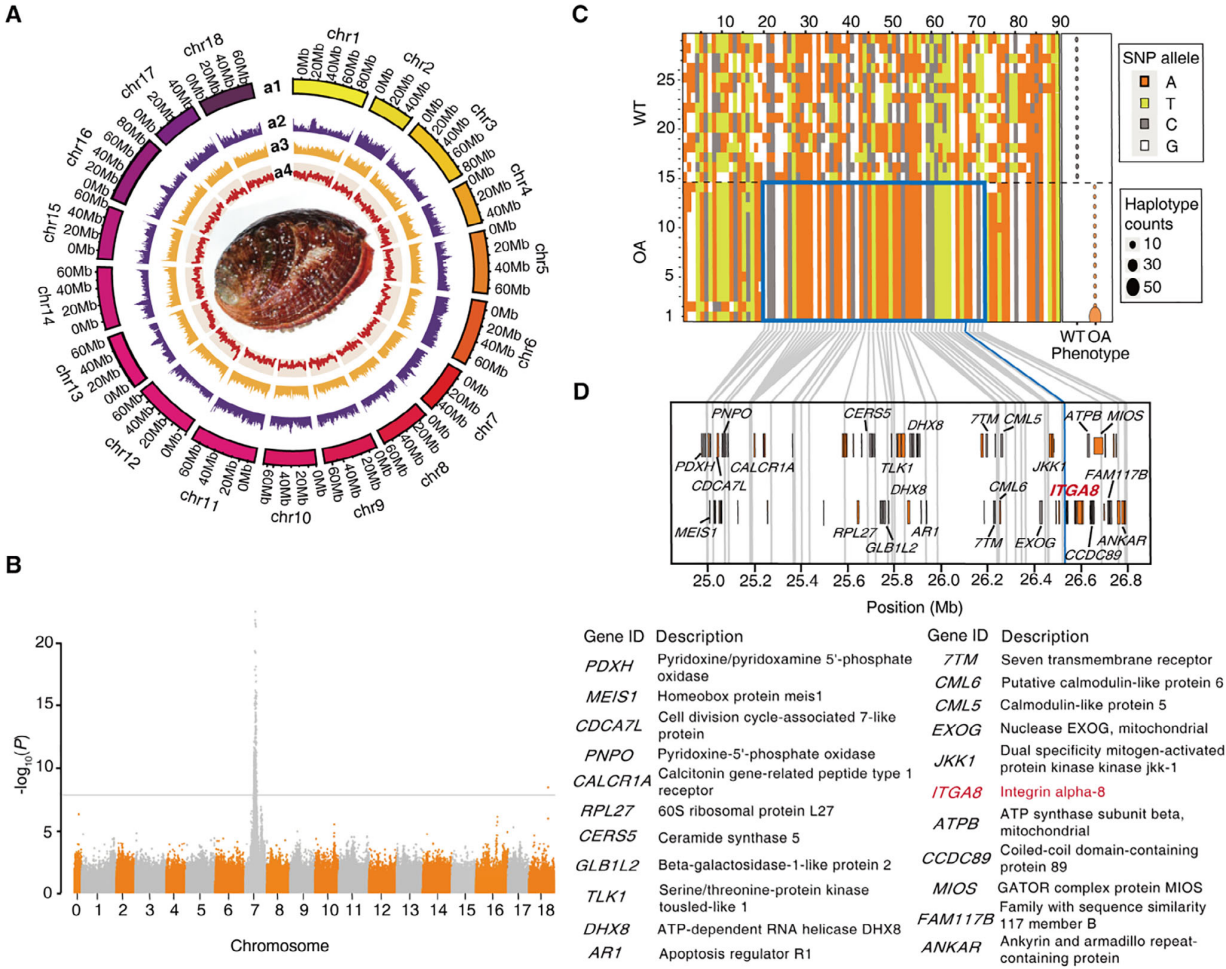
Genomic landscape of *H. gigantea* and genome‐wide association mapping of the orange‐muscle recessive abalone. (A) The genomic landscape of *H. gigantea* is represented in a circular visualization, with four concentric circles conveying different information throughout the genome: a1: 18 Chromosomes at 1‐Mb scales; a2: Density of repeats; a3: GC density; a4: Gene density. (B) Manhattan plots of the orange‐muscle locus. *P*‐values are shown for 5 671 445 SNPs segregating from 100 *H. gigantea*. The line represents the genome‐wide significance threshold (−log_10_(1e‐08) = 8). (C) Haplotypes associated with the orange muscle phenotype. Haplotypes for 225 significant SNPs (*P*‐value < 1e‐8) around rs26532113 with a pairwise r^2^ > 0.5 on the orange‐muscle locus. Association peaks were inferred using the PHASE software program. Almost all orange‐muscle individuals shared a single haplotype (1.80 Mb) around rs26532113, bounded by SNPs rs24997956 and rs26797726. (D) RefSeq gene models and descriptions of genes located within the orange‐muscle haplotype, with the positions of SNPs shown. The blue line indicates the location of SNP rs26532113. The bars within the black box represent the exons of each gene, with different colors used to distinguish the different genes.

To gain insights into the genetic factors involved in carotenoid accumulation in orange‐muscle abalone, we sequenced and assembled a high‐quality reference genome of *H. gigantea*. The genome size of *H. gigantea* was estimated to be 1.43 Gb based on the k‐mer method, with a heterozygosity level of 0.80% (Figure S3A). Employing nanopore sequencing technology with long reads (157 Gb, ∼110 ×) (Table S1), we generated a chromosome‐level genome assembly with the assistance of Hi‐C reads (Figure S3B). The final *de novo* genome assembly of *H. gigantea* spans approximately 1.30 Gb, with contig N50 and scaffold N50 lengths of 2.0 and 73.4 Mb, respectively (Table S2). Nearly 98% of the genome was anchored to the 18 chromosomes (Figure 2A). To assess the completeness of the genome assembly, we conducted Core Eukaryotic Gene Mapping Approach (CEGMA) analysis, which revealed a completeness score of 92.74% (Table S3). Furthermore, our exploration of Benchmarking Universal Single‐Copy Orthologs (BUSCO) indicated that our assembly contained complete sequences for 95.4% of the 978 conserved genes, with only 3.1% missing (Table S4). To evaluate the overall quality and integrity of the assembly, approximately 96.16% of the Illumina short reads were well aligned to the assembly (Table S5). These results strongly support the high quality of our genome assembly. The repeats occupied over half of the *H. gigantea* genome (59.60%), similar to observations reported in other mollusk genomes (Table S6). Notably, transposable elements (TEs) constitute approximately half of the genome, with DNA transposons and LINE elements contributing to 22.84% and 13.50% of the genome, respectively (Table S7). In total, we successfully generated 29,874 protein‐coding gene models, 99.2% of which were annotated based on multiple databases (Figure S3C). This high‐quality, chromosome‐scale genome assembly provides a solid foundation for investigating the underlying mechanisms of color polymorphism in abalone.

### 2.3 Identification of the Gene Responsible for Color Polymorphism in Abalone

To identify the gene locus responsible for the orange muscle, which is rich in carotenoids, in the giant abalone, we conducted a genome‐wide association study (GWAS) based on SNPs identified through whole‐genome resequencing (WGS). DNA was extracted from the muscles of 100 farmed abalone, including 50 orange‐muscle and 50 wild‐type abalone. After removing high missing rates (> 90%) and low‐frequency variants (minor allele frequency < 5%), a total of 5 671 445 SNPs remained. On average, the marker density was 222 bp/SNP across the whole genome. For the linkage disequilibrium (LD) analysis, the overall mean r^2^ between syntenic marker pairs was 0.06. A maximum average r^2^ of 0.68 was estimated for SNPs less than 10 bp apart. However, this value declined rapidly to 0.1 at a distance of around 2 kb (Figure S3D). Considering the average count of 18 SNPs in 4‐kb bins, almost all trait loci would exhibit LD with at least one of these SNP markers.

The GWAS revealed that a single region on chromosome 7 was significantly associated with pigmentation differences between wild‐type and orange‐muscle abalone (Figure 2B; Figure S3E). The risk SNP rs26532113 exhibited the highest degree of linkage to the trait (*P* = 1.47e‐23) (Figure 2B). A total of 225 significant SNPs (*P* < 1e‐8) around rs26532113 with a pairwise r^2^> 0.5 remained for further analysis. To characterize the bounds of the region containing the causative variant, we performed haplotype inference for each individual using the PHASE software. The results illustrated that all orange‐muscle individuals shared a single haplotype (1.80 Mb) around rs26532113, which was bounded by the SNPs rs24997956 and rs26797726 (Figure 2C). Additionally, Haplotype 1 exhibited the highest frequency by far, indicating that it may be the ancestral haplotype in orange‐muscle individuals from which all others arise. Assuming a single‐ancestor model, the possible causative variant must be located within this region, which harbors 38 candidate genes (Figure 2D).

Because carotenoids in abalone are directly derived from feeding without transformation, we hypothesized that enhanced absorption or transportation function may contribute to high carotenoid enrichment based on previous research [29]. We searched for evidence in the literature that any of these 38 genes might contribute to carotenoid or lipid absorption and transportation functions. Integrin subunit alpha 8 harbors rs26532113 and is thought to regulate cell interactions, cell adhesion, and signaling pathways, possibly playing a role in tissue homeostasis and the transport of external substances, such as nutrients, inside and outside cells [30, 31, 32], and is considered the most promising candidate gene.

### 2.4 Differential Expression of *ITGA8* Isoforms in Wild‐Type and Orange‐Muscle Abalone

**FIGURE 3:**
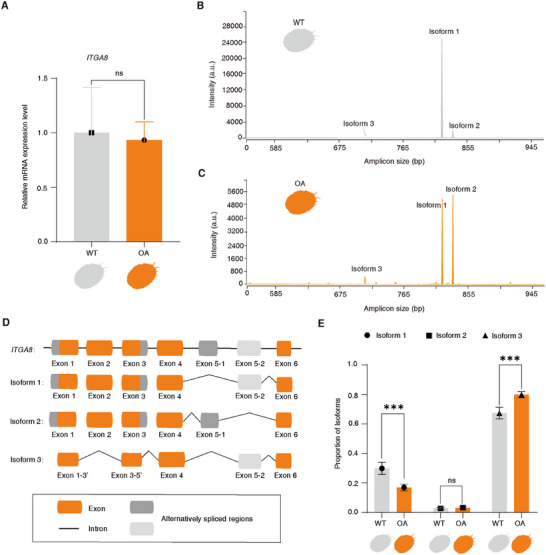
*ITGA8* isoforms and their relative expression in muscles from wild‐type and orange‐muscle abalone. (A) The expression of *ITGA8* in the muscles of wild‐type and orange‐muscle abalone was compared by qPCR using primers targeting the sequence shared by all three isoforms. Data are expressed as the mean ± SD after being calculated relative to the control gene *ICG1* (n = 5). NS: Not significant for OA vs. WT; Student's *t*‐test. (B) and (C) Three *ITGA8* isoforms were found in wild‐type (B) and orange‐muscle (C) abalone, according to capillary electrophoresis analysis of *ITGA8* cDNA fragments across exons 1–11 in the muscles (n = 3). The intensity of the peaks in this multitemplate amplification is biased toward short amplicons; therefore, the isoform abundance may not always be represented by peak intensity. a.u.: Arbitrary units. (D) Diagrammatic illustration of the three *ITGA8* isoforms. The cDNA of abalone muscle tissue was used to amplify and sequence different PCR products. Primers covering exons 1–11 of the mature isoforms were used for PCR. Exons 1–6 involved in the alternative splicing process are shown. Isoforms 1 and 2 differ in exon 5, which is a mutually exclusive exon. Compared to isoform 1, isoform 3 skips the entire exon 2 and misses the 5′ end of exon 1 and the 3′ end of exon 3. (E) The relative expression proportions of the three *ITGA8* isoforms in the muscles of wild‐type and orange‐muscle abalone (n = 5). Data are expressed as the mean ± SD after being normalized to the sum of the three isoforms, which was calculated relative to the common sequence of *ITGA8*. Statistical analyses: Student's *t*‐test. ^***^ *P* < 0.001; NS: Not significant.

Considering that *ITGA8* is most likely associated with the orange muscle recessive phenotype, we first examined its mRNA expression levels using RT‐qPCR analysis in both the muscles of orange‐muscle and wild‐type abalone. The results showed no significant difference in the overall expression of *ITGA8* (Figure 3A). Given the possibility of alternative splicing, we proceeded to examine the *ITGA8* isoforms using RT‐PCR and capillary electrophoresis. The results identified three major isoforms of *ITGA8* in the abalone muscle, which were sequenced by Sanger sequencing (Figure 3B,C; Figure S4). It was found that isoform 1 and isoform 2 differ in exon 5, a mutually exclusive exon (MXE). Compared to isoform 1, isoform 3 skips the entire exon 2 and lacks the 5′ end of exon 1 and the 3′ end of exon 3 (Figure 3D). The prediction of protein tertiary structure indicates that the different *ITGA8* isoforms result in changes in the tertiary configuration of the β‐propeller, which affect the binding of *ITGA8* to ligands, thus changing their activities and functions. In particular, the integrin β‐propeller's classical structure is partially lost in the protein tertiary structure encoded by isoform 3, which negatively impacts the protein's activity and function (Figure S5A–D). Interestingly, the capillary electrophoresis results showed that the intensity of isoforms 1 and 3 in wild‐type muscle differed from that in orange muscle, whereas isoform 2 remained unchanged (Figure 3B,C). Next, qPCR analysis was applied to quantify the relative expression of each isoform, revealing that the proportion of isoform 1 in orange muscle was significantly lower than that in wild‐type muscle (*P* < 0.01), while the proportion of isoform 3 in orange muscle was significantly higher than that in wild‐type muscle (*P* < 0.01). The proportion of isoform 2 was not significantly different between the two groups (*P* > 0.05) (Figure 3E).

### 2.5 The rs26532113 Regulates the Alternative Splicing of *ITGA8*

**FIGURE 4:**
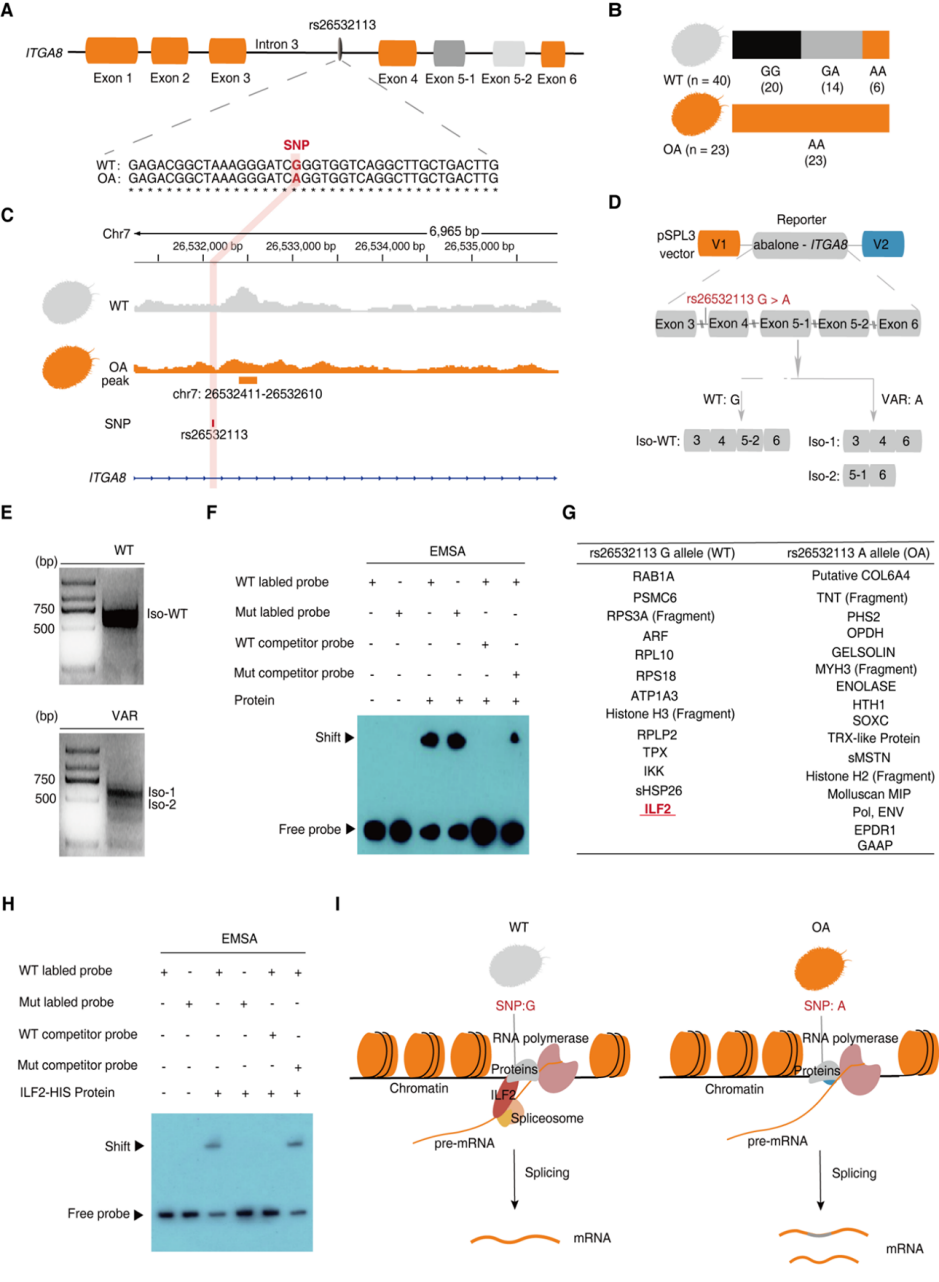
Functional verification of rs26532113 regulating the alternative splicing. (A) The position of rs26532113 is located in the deep area of intron 3 of *ITGA8* in *H. gigantea*. (B) The phenotype‐to‐genotype matching of rs26532113 using 63 individuals of known phenotype is shown. Sample sizes for each genotype in the two groups are marked under the bars. (C) ATAC‐seq accessibility at genomic loci surrounding rs26532113 in wild‐type and orange‐muscle abalone groups. The light pink box indicates the location of rs26532113. The orange solid box represents the ATAC‐seq merged peak. (D) and (E) Schematic design of a minigene assay for alternative splicing using different rs26532113 genotypes (D) and RT‐PCR electrophoresis of the SNP‐reporters with different genotypes (E). Synthetic DNA fragments containing abalone *ITGA8* exons 3, 4, 5‐1, 5‐2, and 6 and the shortened corresponding introns (preserving wild‐type and SNP variation) were assembled into the intron of the pSPL3 vector. WT: wild‐type; VAR: SNP variation; Iso‐WT: Isoform observed in the wild‐type minigene, including *ITGA8* exons 3, 4, 5‐2, and 6; Iso‐1: Isoform observed in the variant minigene, including *ITGA8* exons 3, 4, and 6; and Iso‐2: Isoform observed in the variant minigene, including *ITGA8* exons 5‐1 and 6. (F) DNA‐EMSA validation of binding proteins around different genotypes of rs26532113. (G) List of differential binding proteins for wild‐type and variant genotypes identified by mass spectrometry according to the DNA pull‐down assay. (H) EMSA experiment of different SNP genotypes of DNA probes binding to purified ILF2 protein. (I) Schematic representation of how different SNP genotypes regulate alternative splicing between wild‐type and orange‐muscle abalone. The rs26532113 variation disrupts the interaction between *ITGA8* pre‐mRNA and the splicing factor ILF2, thereby regulating *ITGA8* alternative splicing in orange‐muscle abalone.

Given the potential importance of *ITGA8* in the colorization process of abalone muscles, particularly its alternative splicing alterations observed in abalone muscles with different colors, it is noteworthy that the risk‐associated SNP rs26532113 is situated within intron 3 of *ITGA8* (Figure 4A). We hypothesized that this SNP might affect the alternative splicing of *ITGA8*. We first confirmed that all orange‐muscle recessive individuals were homozygous for the intron variation (“A”), whereas most wild‐type individuals were mainly homozygous or heterozygous for the wild‐type allele (“G”) (Figure 4B; Figure S4A,B). A limited number of wild‐type phenotypic and genotypic mismatches may result from phenotypic mismatches or other genetic or environmental factors, indicating the possibility of unidentified genetic or environmental involvement. Importantly, whole‐genome alignment of different abalone species revealed that this SNP variant occurs in an evolutionarily conserved intronic region of abalone (Figure S6C). We then evaluated whether rs26532113 is associated with *ITGA8* alternative splicing. To this end, we included parts of the DNA sequences from exons 3–6 of abalone *ITGA8*, both wild‐type and variant SNPs, in two minigene reporters (Figure 4D; Figure S6D). The reporters were then stably expressed in HEK293T cells, and one single band (isoform) was observed in the wild‐type minigene, whereas two bands (isoforms) were observed in the minigene containing the variant SNP (Figure 4E). According to Sanger sequencing, the alternative splicing types in the mutant reporter included mutually exclusive exons (MXE) and skipped exons (SE) (Figure 4E; Figure S6E).

After determining that rs26532113 can regulate *ITGA8* alternative splicing, we sought to explore the underlying molecular mechanisms. Transposase‐accessible chromatin sequencing (ATAC‐seq) was conducted to detect chromatin accessibility in the abalone muscle to assess the functional potential of the genomic region surrounding this SNP. The fact that ATAC‐seq peaks were found approximately 298 bp downstream of this SNP location in both wild‐type and orange‐muscle abalone indicates that rs26532113 is situated in a regulatory domain (Figure 4C; Figure S7A). We then examined whether the genomic region where the rs26532113 SNP is located has any protein‐binding capacity using EMSA analysis, and if so, whether the rs26532113 SNP has any effects on such capacity. According to the EMSA results with the 24‐bp double‐stranded oligonucleotide probes, including different genotypes of rs26532113, the proteins associated with genomic sequences of wild‐type and variant rs26532113 differed significantly (Figure 4F). We then conducted DNA pull‐down assays to identify the proteins that bind to each genotype. The silver staining and mass spectrometry analysis results demonstrated distinct binding proteins with probes from genomic sequences of wild‐type and variant rs26532113 (Figure 4G; Figure S7B,D, Tables S8 and S9). Importantly, we discovered a splicing factor, ILF2 (Interleukin enhancer‐binding factor 2), which belongs to the family of proteins with conserved double‐stranded DNA‐binding and RNA‐binding domains in wild‐type abalone (Figure 4G). Next, the ILF2 protein was purified (Figure S7E), and an EMSA experiment was conducted with the 24‐bp double‐stranded oligonucleotide probes at which the SNP was located. The EMSA experiments of different genotypes of DNA probes binding to ILF2 confirmed the selective affinity of ILF2 toward the rs26532113 G allele probe, with no binding capability toward the rs26532113 A allele probe (Figure 4H). Studies have shown that ILF2 can bind not only DNA but also a variety of RNA‐binding proteins (RBPs), including nucleophosmin (NPM), Y‐box binding protein 1 (YB‐1), nucleolin, adenosine deaminase RNA specific (ADAR1), and various heterogeneous nuclear ribonucleoproteins (hnRNPs), and directly regulate the alternative splicing and stability of specific pre‐mRNAs [33]. Considering this information, we deduce that rs26532113 variation may result in altered binding proteins, particularly in the case of ILF2 deletion in orange‐muscle abalone, altering its interaction with the spliceosome and the *ITGA8* splicing patterns (Figure 4I).

### 2.6 Multi‐Omics Profiling Reveals Tissue Remodeling in Orange Muscle

**FIGURE 5:**
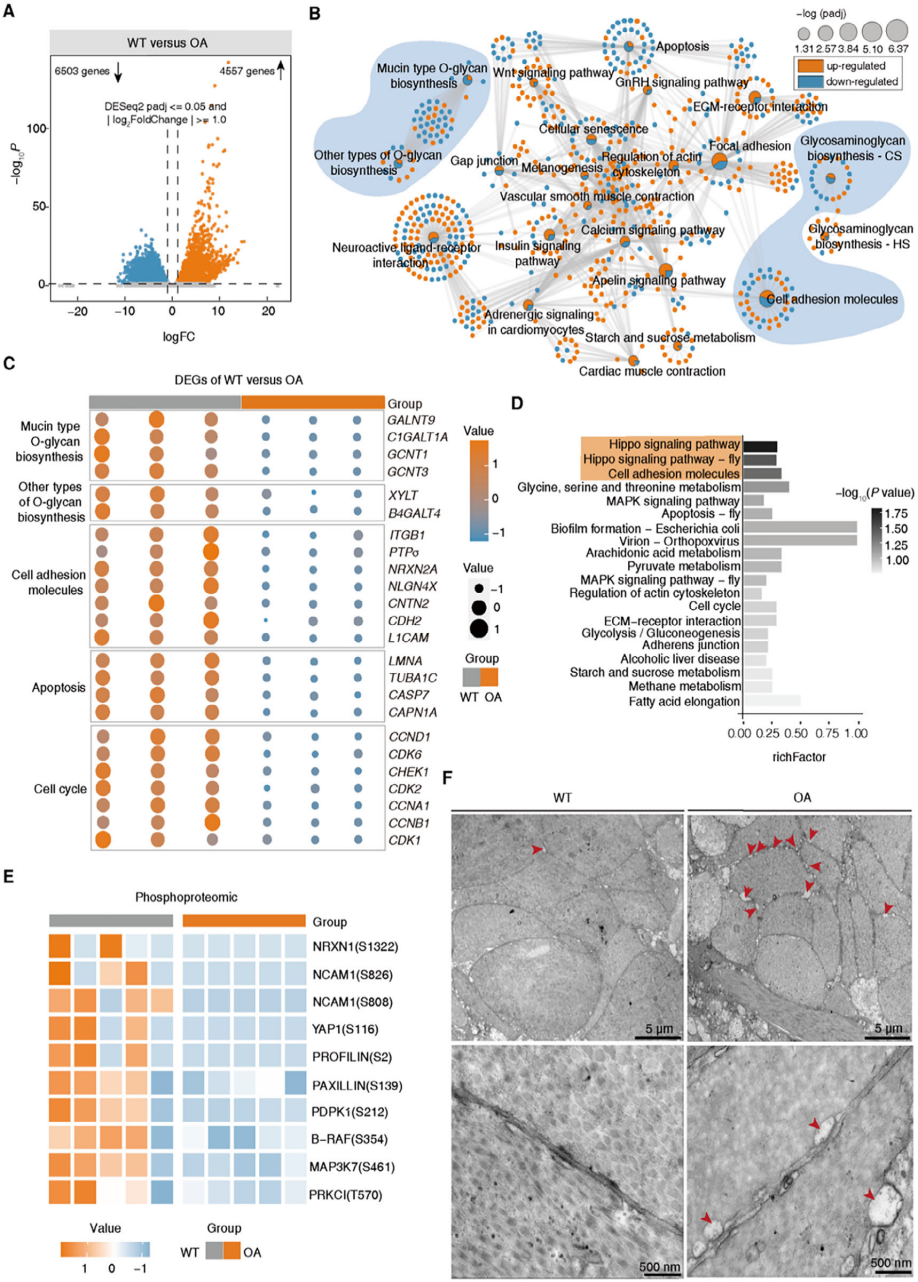
Multi‐omics analysis between wild‐type and orange‐muscle abalone muscles and the role of tissue cell architecture in carotenoid accumulation in abalone. (A) The volcano map of the expression of the differentially expressed genes in orange muscle compared to wild‐type muscle. Genes passing both thresholds (DESeq2 padj ≤ 0.05 and |log_2_FoldChange| ≥ 1.0) are colored. (B) Network visualization of significantly enriched KEGG pathways between wild‐type and orange muscles by transcriptome analysis. Network nodes are sized by adjusted *P* value. Up‐regulated genes are represented by orange dots, and down‐regulated genes are represented by blue dots. The light blue‐highlighted pathways represent significantly enriched pathways for down‐regulated genes in orange muscle. (C) Heatmaps of representative genes changes in related significant enrichment pathways identified in wild‐type and orange muscles by transcriptome analysis. (D) The enriched KEGG pathways of down‐regulated phosphopeptides in orange muscles. The light orange‐highlighted pathways represent significantly enriched pathways for down‐regulated phosphopeptides. (E) Heatmaps of representative phosphopeptides changes were identified in wild‐type and orange muscles. (F) Representative TEM images showing changes in the tissue architectures of wild‐type and orange muscle samples (n = 3). Red arrow: Enhanced intercellular spaces. Scale bars: 5 µm (top row) and 500 nm (bottom row).

To elucidate the specific mechanisms underlying the differences in carotenoid accumulation between wild‐type and orange‐muscle abalone, we evaluated the transcriptional characteristics and protein phosphorylation changes in muscles of different colors. A total of 4557 upregulated genes and 6503 downregulated genes were identified in orange muscle compared to wild‐type muscle, according to the transcriptome analysis (Figure 5A; Table S10). Functional enrichment analysis of the differentially expressed genes (DEGs) in different color abalone muscles showed that integrin‐mediated pathways were significantly enriched, including focal adhesion, cell adhesion molecules, ECM‐receptor interaction, and regulation of actin cytoskeleton (Figure 5B) [34]. Specifically, upregulated genes were significantly enriched in pathways related to ECM‐receptor interaction, focal adhesion, regulation of the actin cytoskeleton, and others (Figure S8A); conversely, downregulated genes exhibited significant enrichment in cell adhesion molecules, mucin‐type O‐glycan biosynthesis, other types of O‐glycan biosynthesis, glycosaminoglycan synthesis‐CS, as well as processes associated with cell apoptosis and cell cycle (Figure 5B,C; Figure S8B). Remarkably, the genes associated with cell adhesion molecules, mucin‐type O‐glycan, and glycosaminoglycan synthesis, which are involved in cell‐cell adhesions, represented a strongly and significantly downregulated subgroup in orange muscle. Most of the genes related to O‐glycan synthesis (such as *GALNT9*, *C1GALT1A*, *GCNT1*, and *XYLT*) were found to be downregulated in orange muscle, along with several genes within the cell adhesion molecules, including *ITGB1*, *NRXN2A*, *NLGN4X*, *CDH2*, *CNTN2*, and *L1CAM* (Figure 5C).

The 4D label‐free phosphoproteomic analysis revealed that, compared to wild‐type muscle, 38 phosphopeptides were upregulated and 102 were downregulated in orange muscle (Figure S8C; Table S11). Additionally, 116 phosphopeptides were exclusively expressed in orange muscle, while 183 were uniquely present in wild‐type abalone muscle (Table S12). The downregulated phosphopeptides were significantly enriched in cell adhesion molecules and the Hippo signaling pathway (Figure 5D). The phosphorylation levels of proteins associated with cell adhesion molecules, such as NRXN1 and NCAM1, were downregulated in orange muscle. Additionally, the phosphorylation levels of proteins involved in integrin‐mediated signaling transduction, including PAXILLIN, PDPK1, B‐RAF, MAP3K7, and YAP1, were also downregulated in orange muscle (Figure 5E). It is well known that when activated, integrins trigger multiple downstream kinases that transduce signals by activating the phosphorylation of multiple protein tyrosine or serine kinases [35]. Together, these data suggest that integrin‐mediated pathway networks play a crucial role in the differences between abalone muscles of different colors. They provide evidence for the remodeling of cell‐cell adhesion in orange muscle, which is associated with integrin‐mediated signal transduction.

According to related research, weakened cell‐cell adhesion results in widened intercellular spaces and increased tissue permeability to lipoproteins and other molecules [20]. Based on these analyses, we speculate that widened intercellular spaces and increased tissue permeability may be the main features of enhanced carotenoid accumulation in orange muscle. To validate our hypothesis, we explored whether the muscle architecture in orange‐muscle abalone was remodeled at the ultrastructural level. Transmission electron microscopy (TEM) analysis showed obvious expansion of the intercellular spaces in orange muscles (Figure 5F; Figure S8D). In addition, the cell membranes of the orange muscle cells were blurred, and the intracellular matrix was sparse in certain localized areas (Figure 5F; Figure S8D). In view of these analyses, we hypothesize that orange‐muscle abalone promotes carotenoid accumulation by increasing tissue permeability, driven by widened intercellular spaces and changed tissue architecture resulting from the remodeling of cell‐cell adhesion, with integrin‐mediated signal transduction playing a crucial role in this process.

### 2.7 Key Roles of *ITGA8* and its Alternative Splicing in Carotenoid Accumulation

**FIGURE 6:**
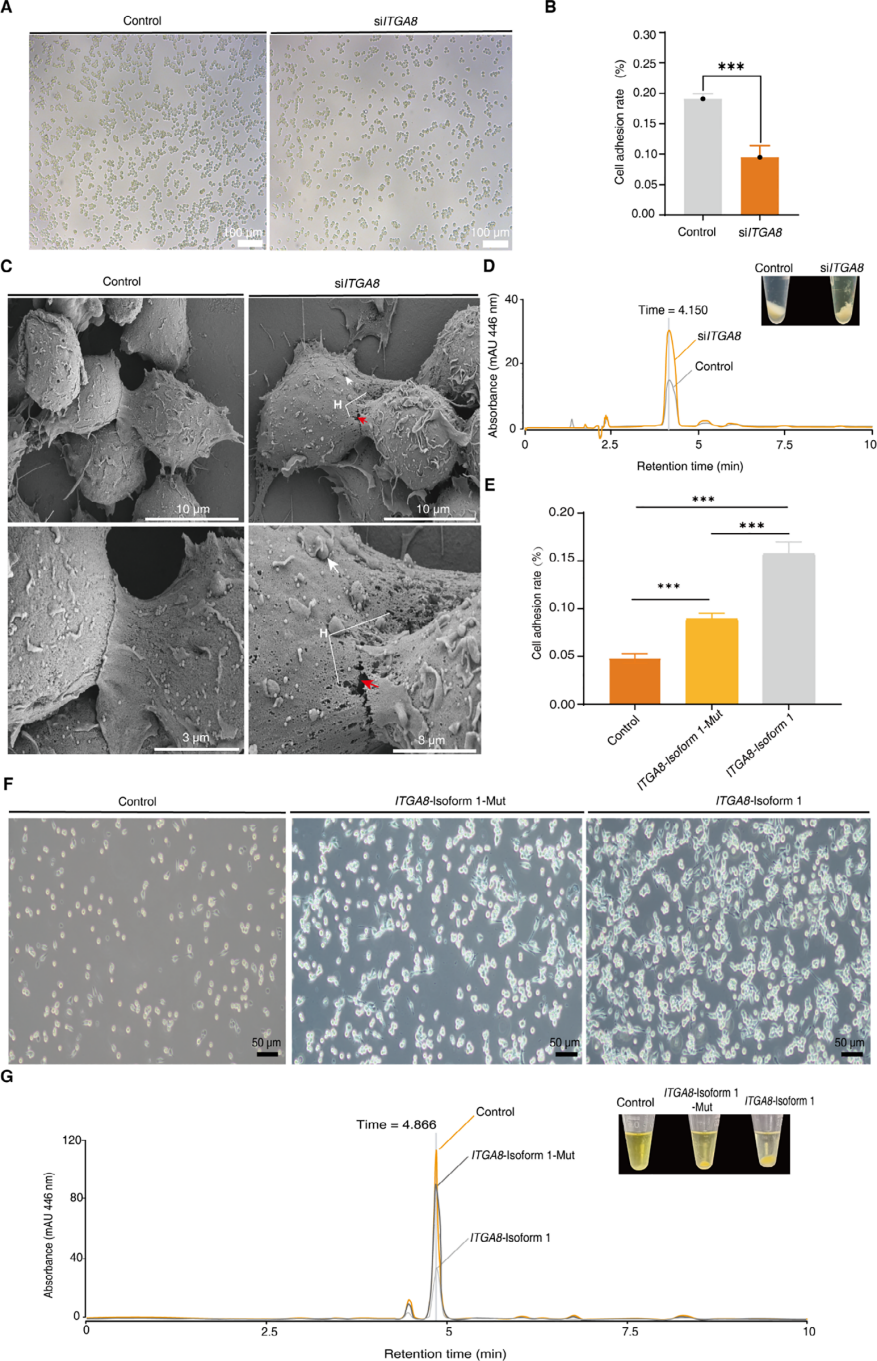
Roles of *ITGA8* and its alternative splicing in carotenoid accumulation in RAW264.7 cells. (A) Representative images of changes in cell adhesion between the control and *ITGA8*‐knockdown groups (n = 3). (B) The cell adhesion rate was significantly decreased in the *ITGA8*‐knockdown group. Statistical analyses: Student's *t*‐test. ^***^ *P* < 0.001. (C) Representative scanning electron microscopy images showing changes in the cell structure of the control and *ITGA8*‐knockdown groups (n = 3). H: Cellular holes; Red arrow: Enlarged intercellular space; White arrow: Raised cell membrane. (D) Normal‐phase HPLC chromatogram of zeaxanthin between the control and *ITGA8*‐knockdown groups. Images of absorbed zeaxanthin extract from the *ITGA8*‐knockdown (right) and control (left) groups. (E) The cell adhesion rate was compared among the control, mouse *ITGA8*‐isoform 1‐Mut, and mouse *ITGA8*‐isoform 1 overexpression groups. Statistical analyses: Student's *t*‐test. ^***^ *P* < 0.001. (F) Representative images of changes in cell adhesion among the control, mouse *ITGA8*‐isoform 1‐Mut, and mouse *ITGA8*‐isoform 1 overexpression groups (n = 9). (I) Normal‐phase HPLC chromatogram of absorbed zeaxanthin extract from the control, mouse *ITGA8*‐isoform 1‐Mut, and mouse *ITGA8*‐isoform 1 overexpression groups. Images of absorbed zeaxanthin extract from the control (right), mouse *ITGA8*‐isoform 1‐Mut (middle), and mouse *ITGA8*‐isoform 1 overexpression (left) groups.

The alterations in *ITGA8* alternative splicing patterns and integrin‐mediated pathway networks suggest that the changed *ITGA8* splicing pattern may play a crucial role in carotenoid accumulation. To this end, we first applied small interfering RNA (siRNA) to specifically downregulate *ITGA8* in RAW264.7 cells (Figure S9A). Inhibition of *ITGA8* significantly reduced cell adhesion rates (*P* < 0.001) (Figure 6A,B). Interestingly, the *ITGA8*‐knockdown group demonstrated cell damage, localized holes in the cell membrane, and enlarged intercellular spaces, according to the results of scanning electron microscopy (Figure 6C; Figure S9B). This finding verified that increasing the permeability of the cell membrane, inducing damage to the cell membrane, and expanding the intercellular spaces are all possible outcomes of inhibiting *ITGA8* expression. The decreased *ITGA8* also significantly enhanced the lipid accumulation capacity of cells (Figure S9C,D). Subsequently, we investigated whether elevated carotenoid accumulation was directly boosted by inhibiting *ITGA8* expression. The accumulation of high levels of carotenoids resulted from decreased *ITGA8* expression, as demonstrated by the findings of the zeaxanthin absorption experiment (Figure 6D). These findings confirm the function of *ITGA8* in carotenoid accumulation. When *ITGA8* is inhibited, enlarged intercellular spaces and increased cell permeability can promote carotenoid accumulation.

We then constructed the mouse *ITGA8*‐isoform 1 and the mouse *ITGA8*‐isoform 1‐Mut overexpression plasmids and transfected them into RAW264.7 cells (Figure S11A,B). Sequence alignment and protein tertiary structure prediction revealed that the mouse *ITGA8*‐isoform 1 has similar sequence variations and protein structure to the abalone *ITGA8*‐isoform 1 (Figure S10A–C). Protein tertiary structure prediction indicated that the structure of the protein encoded by the mouse *ITGA8*‐isoform1‐Mut sequence is similar to that of the protein encoded by the abalone *ITGA8*‐ isoform 3, both exhibiting a deletion in a homologous region of the integrin β‐propeller tertiary structure (Figure S10D,E). Overexpression of mouse *ITGA8*‐isoform 1 and *ITGA8*‐isoform 1‐Mut significantly enhanced the cell adhesion rate (*P* < 0.001) and decreased cellular lipid droplet accumulation (*P* < 0.001) and zeaxanthin absorption (Figure 6E–G; Figure S11C–F). Compared to mouse *ITGA8*‐isoform 1, overexpression of *ITGA8*‐isoform 1‐Mut significantly reduced the cell adhesion rate (*P* < 0.001) and increased cellular lipid droplet accumulation (*P* < 0.001) and zeaxanthin uptake (Figure 6E–G; Figure S11C–F). These results indicate that the absence of the corresponding structural element in the β‐propeller tertiary structure of *ITGA8* markedly reduces its integrin function. Overall, these findings imply that *ITGA8* and its alternative splicing can regulate cellular lipid and carotenoid accumulation by modulating cell adhesion.

## 3 Discussion

**FIGURE 7:**
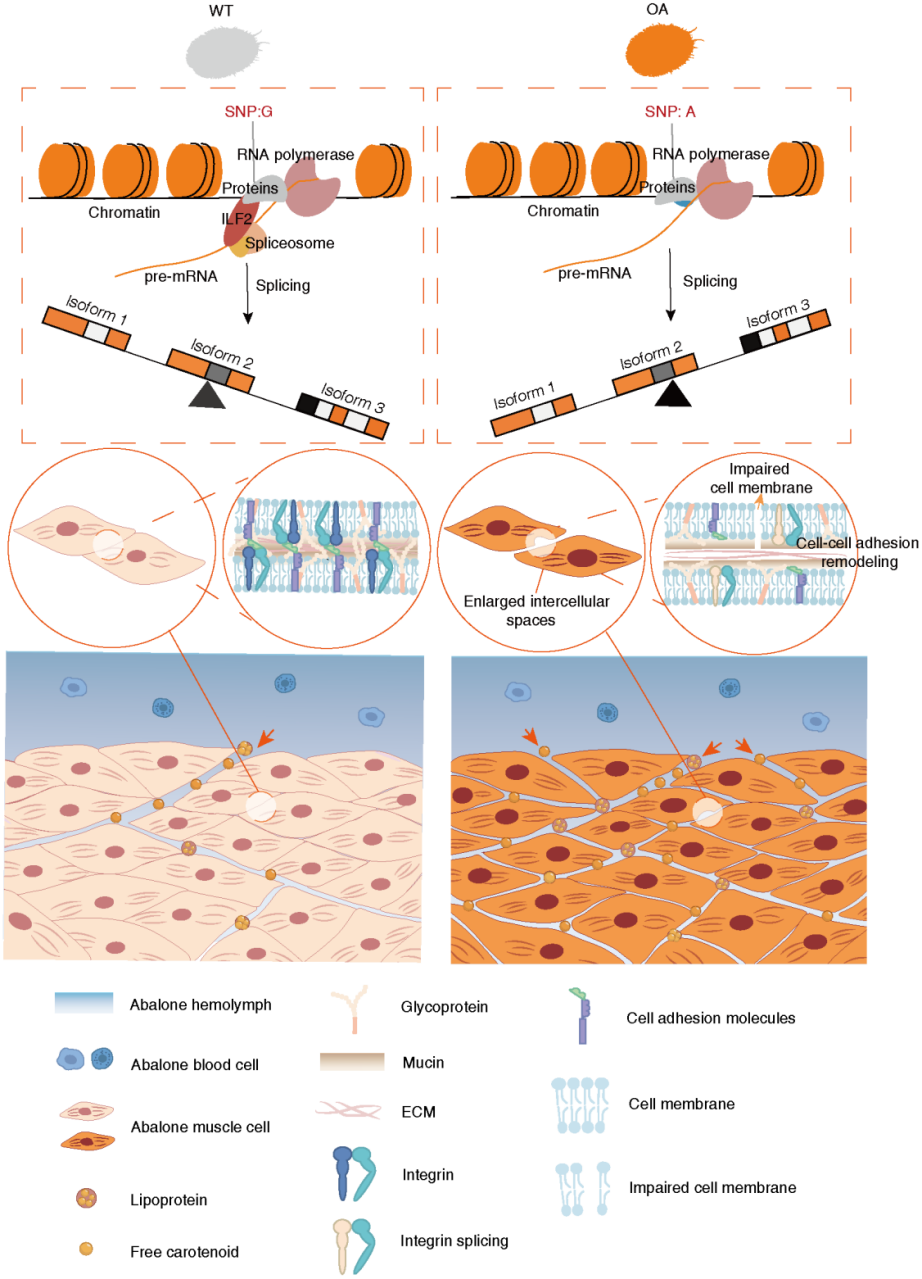
Schematic illustration of the mechanism underlying the accumulation of differential carotenoids between the muscles of wild‐type and orange‐muscle abalone. In wild‐type abalone muscle tissue, cells are tightly arranged, intercellular spaces are normal, and the cell membrane structure is intact. Compared to wild‐type abalone, a single‐nucleotide polymorphism disrupts the interaction between *ITGA8* pre‐mRNA and the splicing factor ILF2, consequently modulating *ITGA8* alternative splicing to remodel intercellular spaces in orange muscle. Specifically, the intercellular spaces in the muscle tissue of orange‐muscle abalone are widened and cell‐cell adhesion is remodeled. The cell membrane appears blurred and impaired. The circulatory carotenoid levels absorbed by orange muscle are increased.

Biological color polymorphism involves a complex formation process, and exploring this process is crucial for enhancing our understanding of the genetics, morphology, growth, and physical mechanisms that contribute to phenotypic diversity [2]. The formation of color phenotypes typically relies on the coordinated integration of regulatory networks spanning chemical, biological, and physical dimensions [2]. However, the extent to which internal structures influence carotenoid‐based color polymorphism, and the multi‐scale coordination among genetic regulation, cellular states, and tissue architecture underlying this phenomenon, remains insufficiently understood. In this study, we identified a non‐coding SNP in the *ITGA8* integrin gene of *H. gigantea* that induces alternative splicing changes, enhancing tissue permeability by widening intercellular spaces. This process is mediated by the remodeling of cell adhesion, thereby promoting carotenoid accumulation (Figure 7).

Although integrins are recognized as key regulators of tissue homeostasis, their potential association with carotenoid coloration in animals remains unreported. In exploring the mechanisms underlying carotenoid coloration in animals, previous research has predominantly focused on the 1D genetic regulation of key genes involved in carotenoid absorption, metabolism, and transport pathways. Studies have primarily examined mutations and expression changes in genes such as *BCO1* (beta‐carotene oxygenase 1), *SCARB1* (scavenger receptor class b, type I), and *CYP2J19* (cytochrome p450 enzyme) [13, 14, 36]. In this study, we identified, for the first time, the important role of *ITGA8* in carotenoid coloration in animals, and confirmed that *ITGA8* is a key gene responsible for carotenoid enrichment in orange‐muscle abalone. Integrins are a family of transmembrane proteins that consist of heterodimers formed by α and β subunits. The extracellular domains of these proteins bind to extracellular matrix proteins or other cell surface molecules, while their cytoplasmic domains interact with cytoskeletal and signaling proteins. Consequently, integrins are uniquely positioned to mediate the transfer of information between the extracellular environment and the intracellular space, facilitating bidirectional cellular communication [37]. Alternative splicing of integrins results in sequence variations in both the extracellular and cytoplasmic domains, serving as a sophisticated mechanism to modulate integrin‐ligand interactions and downstream signaling. Changes in splicing patterns can impact associated biological processes and signaling pathways, thereby influencing cellular functions and responses [37]. Alterations in the alternative splicing patterns of *ITGA8* observed in abalone muscles of varying colors lead to modifications in cellular states and tissue architecture, ultimately contributing to the high accumulation of carotenoids. The *ITGA8* knockdown experiment in RAW264.7 cells provides strong evidence for the role of *ITGA8* in regulating cell adhesion and carotenoid accumulation, while the overexpression of mouse *ITGA8*‐isoform 1 adequately confirms its involvement in these processes. Furthermore, overexpression of mouse *ITGA8*‐isoform 1‐Mut fully confirms that the absence of corresponding structural elements in the β‐propeller tertiary structure encoded by abalone *ITGA8*‐isoform 3 significantly reduces its integrin function and regulates cell adhesion and carotenoid accumulation. The multi‐omics analyses of abalone muscles with varying coloration, combined with subsequent functional validation experiments, offer robust evidence that integrin‐mediated regulatory networks can enhance carotenoid accumulation through the enlargement of intercellular spaces and increased tissue permeability.

Species with different phenotypes may have very similar coding sequences, suggesting that regulatory sequences are the primary drivers of phenotypic variation [38]. Our findings established a model for *ITGA8* alternative splicing driven by an intronic risk SNP, where alternative splicing changes contribute to the remodeling of cell‐cell adhesion, resulting in different carotenoid accumulation capacities. In this model, we confirmed that rs26532113 is a locus closely associated with carotenoid coloration in abalone. Risk SNP variation disrupts the interaction between *ITGA8* pre‐mRNA and the splicing factor ILF2, regulating the *ITGA8* alternative splicing changes between abalone muscles of different colors. Although further investigation is warranted to elucidate the underlying genetic and molecular mechanisms governing alternative splicing at a more profound level, and the existence of other unknown variants associated with this SNP cannot be ruled out, this variant provides important insights for further exploring the causal variation related to carotenoid accumulation in orange‐muscle abalone. Additionally, it offers potential molecular marker insights for developing high‐quality abalone varieties rich in carotenoids.

For the first time, we demonstrate that a non‐coding SNP in the *ITGA8* gene induces alternative splicing, which drives phenotypic variation in carotenoid‐based coloration by modulating endogenous tissue remodeling processes, including cell state transitions and reconstruction of the physical microenvironment. This work underscores the pivotal regulatory role of non‐coding variants in trait formation and offers critical insights into the complex genotype‐phenotype relationship. Previous studies on carotenoid‐based color polymorphism have primarily focused on the genetic regulatory roles of key genes. However, the precise mechanisms by which genetic variation modulates cellular state transitions to remodel tissue architecture, thereby jointly driving carotenoid‐based color polymorphism, remain poorly understood. The relationship between genes and phenotypes is not a simple linear one, and 1D gene studies are insufficient to fully reveal the complexity of phenotypes. Relevant studies have shown that genetic mechanisms act in concert with generic physical mechanisms, such as adhesion, surface tension, and phase separation, to collectively drive the final morphology of multicellular organisms [24]. Within complex cell‐matrix systems, diverse adhesive interactions are pervasive, and weak adhesion may disrupt mechanical equilibrium, thereby promoting the remodeling and evolution of tissue structure and morphology [24]. Our discovery addresses a previously unrecognized gap in our understanding of the interplay between genetic and structural factors underlying carotenoid‐based coloration in animals, and lends empirical support to the earlier hypothesis suggesting an integration of carotenoid uptake and feather development in birds [39]. It's well established that in vertebrates such as fishes, amphibians, and reptiles, color patterns arise from the spatial arrangement of chromatophores [40]. However, whether spatial structural changes driven by alterations in animal tissue cell states contribute to pigment‐based coloration only in species‐specific cases of carotenoid‐based polymorphism, or more broadly influence pigment deposition across taxa, including mollusks and even vertebrates, remains to be elucidated. Investigating how genetic mechanisms interact with cell states and associated physical processes to shape color polymorphism requires in‐depth study. Systematic research into this process will not only enhance our understanding of the mechanisms underlying biological color polymorphism, but may also reveal related principles governing the formation of biodiversity, morphological innovation, and morphological convergence across species.

Alterations in the internal tissue architecture of organisms not only influence pigment deposition but also exert effects on their growth. Genes in the cell cycle were significantly downregulated by transcriptome analysis, and a year‐long culture experiment revealed that orange‐muscle abalone exhibit growth suppression [29]. Moreover, transmission electron microscopy of abalone muscles, combined with results from mouse cell experiments, revealed that reduced cell adhesion induces cell membrane damage and disrupts lipid metabolism. Carotenoid‐based color polymorphism plays an important role in reflecting individual quality; however, the link between carotenoid concentration and individual mass remains a source of debate [41, 42]. Our research reveals the correlation between carotenoid coloration and metabolic growth processes induced by changes in the tissue organization of abalone, providing a clue for elucidating the complex relationships between carotenoid color polymorphism and individual traits in other animals. In particular, this may highlight the important role of carotenoid‐based coloration mechanisms in the process of honest signaling, reflecting individual quality. Understanding the relationships among morphological structures, chemical composition, and biological functions has long been one of the central goals of physiology [2]. Our findings reveal the complex associations between changes in cellular and tissue structures and growth, physiological function, and color polymorphism in animals, offering new perspectives for elucidating the mechanisms underlying biological adaptation.

## 4 Conclusions

This study reveals a previously unrecognized mechanism underlying animal carotenoid pigmentation, providing a multi‐level perspective that integrates genetic variation, alternative splicing, cellular remodeling, and tissue architecture. It elucidates how molecular, physical, and chemical factors synergistically drive the formation of morphological diversity. Our study advances the understanding of how genetic variations influence phenotypic traits through coordinated molecular and cellular programs by elucidating the complex genotype‐phenotype relationships shaped by multiscale regulatory processes. This not only deepens our comprehension of the mechanisms underlying biological phenotypic diversity but also provides new insights into the intrinsic processes driving its emergence and evolution. These findings also identify key molecular targets for cultivating carotenoid‐rich abalone, paving the way for precision aquaculture. More broadly, this study provides new insights into the genetic regulation of complex traits and holds significant implications for functional genomics research across various species. Additionally, our research reveals a novel mechanism by which organisms absorb external substances through tissue cell rearrangement, laying the foundation for the development of future innovative drug delivery systems.

## 5 Methods

### 5.1 Analysis of the Major Carotenoid Contents in Abalone

Normal‐phase high‐performance liquid chromatography with a silica gel column (YMC‐Pack SIL column) (250 × 4.6 mm, 5 µm, 12 nm, YMC Co., Ltd., Kyoto, Japan) was used to determine the primary carotenoid contents of abalone. The mobile phase consisted of n‐hexane (A) and ethyl acetate (B). A linear gradient was used to elute step by step: 1) 40% A and 60% B for 10 min, 2) 100% A for 5 min, and 3) 40% A and 60% B were balanced for 5 min. Elution occurred in 20 min total. The column temperature was 25°C, the detection wavelength was 446 nm, the injection volume was 20 µL, and the flow rate was 1.5 mL/min. The concentrations of zeaxanthin and β‐carotene were determined using standard curves established by Thermo Scientific UltiMate 3000 high‐performance liquid chromatography (Thermo Fisher Scientific, MA, USA), and every group was detected in three replicates. The procedures and protocols used in this research were ethically reviewed and approved in accordance with the guidelines of the relevant institutional committees.

### 5.2 Genome Assembly and Annotation

An adult *H. gigantea* was collected from a Fuda abalone farm located in Fujian Province, China.

Following collection, the individuals were carefully dissected, and the extracted tissues were preserved at ‐80°C using liquid nitrogen to ensure the preservation of DNA and RNA integrity for subsequent sequencing analyses. High‐molecular‐weight (HMW) DNA was extracted from a single female using a Qiagen Genomic Tip DNA Isolation kit (Cat. Nos. 10 243 and 19 060, Qiagen, Hilden, Germany) according to the manufacturer's protocol. Subsequently, Oxford Nanopore Technologies (ONT) libraries were prepared from the extracted HMW DNA, adhering to the guidelines provided for the SQKLSK109 library preparation kit from Oxford Nanopore (Oxford Nanopore Technologies, Cambridge UK). The ONT libraries were then subjected to sequencing using the MinION sequencing device and the ONT MinKNOW Software (Oxford Nanopore Technologies, Cambridge, UK). Specifically, one paired‐end Illumina sequence library and one Hi‐C library with an insert size of 350 bp were constructed using the same extracted DNA and sequenced on the Illumina HiSeq X platform (Illumina, USA). Total RNA was isolated from various tissues of *H. gigantea*, including the muscle, gill, mantle, and hepatopancreas, using a Qiagen RNeasy kit (Qiagen). Total RNA was used for RNA sequencing (RNA‐seq) on an Illumina HiSeq platform. We assembled the *H. gigantea* genome by integrating ONT long‐read sequences, Illumina short‐read sequences, and data from high‐throughput chromatin conformation capture (Hi‐C) technologies. Initially, 157 Gb (∼110×) of high‐quality long reads were used to assemble heterozygous genome sequences using the wtdbg (ver 2) program [43]. To enhance the accuracy of the ONT sequencing data, we generated approximately 142 Gb (∼99×) of Illumina short reads from the same individual. These short reads were mapped against the initial wtdbg genome assembly using BWA (ver 0.7.8) [44] with default parameters. Variants suspected to result from sequencing errors were meticulously refined using Pilon (ver 2) [45]. Subsequently, we assessed the assembly quality using the Core Eukaryotic Gene Mapping Approach (CEGMA) (ver 2.5) [46] and BUSCO (ver 4) [47] completeness and duplication scores. The resulting non‐redundant contigs were subjected to lachesis (ver 201701) [48], which facilitated their partitioning into 18 groups, corresponding to 18 pseudo‐chromosomes. In parallel, repetitive sequences within the genome were annotated by integrating homologous alignment methods with ab initio prediction techniques. We performed the annotation of repeats using tools such as repeatmasker and repeatproteinmask (ver 4.05) [49], LTR_finder (ver 1.07) [50], repeatscout (ver 1.05) [51], and repeatmodeler (ver 1.04) [52]. To annotate protein‐coding genes, we followed a methodology similar to that employed for the nautilus genome [53]. Briefly, we integrated evidence from the transcriptomes of four tissues (foot muscle, gill, mantle, and hepatopancreas) using Trinity (ver 2) [54]. Additionally, we leveraged orthologous proteins from various species, including *Nautilus pompilius* [53], *Lottia gigantea* [55], *Crassostrea virginica* [56], *Pinctada fucata martensii* [57], *Bathymodiolus platifrons* [58], *Mizuhopecten yessoensis* [59], *Drosophila melanogaster* [60], *Branchiostoma floridae* [61], *Caenorhabditis elegans* [62], and *Helobdella robusta* [55]. Finally, ab initio gene prediction was performed using the evidencemodeler (ver 1.1.1) software [63]. To gain insights into the biological functions of selected genes, we conducted GO and KEGG enrichment analyses using R clusterProfiler [64]. Significant enrichment was determined using Fisher's exact test, with *P*‐values adjusted using the Benjamini–Hochberg multiple‐hypothesis‐testing correction.

### 5.3 SNP Calling, LD Estimation, Genome‐Wide Association Analysis, and Haplotype Inference

Fifty *H. gigantea* samples with wild‐type morphology and 50 individuals with orange‐muscle morphology were collected from the Fuda abalone farm. Foot muscle tissues were carefully dissected for DNA extraction and were subsequently sequenced using the Illumina sequencing platform. Sequencing was performed using an average depth of 4X coverage. Sequencing reads were removed using in‐house Perl scripts with the following criteria: 1) aligned to the barcode adaptor; 2) with ≥ 10% unidentified nucleotides (N); and 3) where > 50% of all bases had Phred quality scores ≤ 20. All remaining reads were mapped to the reference genome using BWA (ver 0.7.17) [65] and linearized, sorted, reduplicated, and indexed using SAMtools (ver 1.11) [66] under the default settings. SNPs were detected using GATK (ver 4.0) [67], and hard filtration was conducted using “VariantFiltration” (QD < 2.0; MQ < 40.0; FS > 60.0; SOR > 3.0; MQRankSum < –12.5; ReadPosRankSum < –8.0). Raw SNPs were further quality controlled by filtering using VCFtools (ver 4.2) [68] with the following parameters: –maf 0.05 –max‐missing 0.9 –hwe 0.001. A total of 5 671 445 high‐quality SNPs remained for downstream analysis. The estimation of pairwise linkage disequilibrium (LD) was conducted using PopLDdecay (ver 3.42) [69] with the default parameters. LD decay in 100 samples was analyzed based on nonrandom associations among alleles at different syntenic loci. Bins of 1 kb were created based on the physical distance of each SNP pair. The extent and decay of the LD were visualized by a statistic of the average r^2^ within each bin up to a distance of 300 kb. Files in the input format were transformed from vcf files by PLINK (ver 1.9) [70]. Information on the phenotypes (1 for WT, 2 for OA) was added to. fam file. Considering population genetic diversity, a kinship matrix was constructed using GEMMA (ver 0.98.3) [71] based on the centered genotypes, which has been indicated to be preferable for population structure analyses in lower organisms according to the manual. Genome‐wide association analysis was performed using GEMMA in cooperation with the kinship matrix (−k), while the Wald test was used to calculate the *P*‐value (‐lmm 1). The Bonferroni method was typically employed to calculate the genome‐wide significance threshold of 0.05/n, where n is the number of SNPs used. Here, we adopted a conventional threshold of 1e‐8 to consider the stringent Bonferroni correction. A subset of SNPs in chr7 was extracted for haplotype inference. For interval determination, PLINK was used to calculate the pairwise r^2^ between SNP chr7:26532113, which has the highest association with the phenotype, and those around it. All SNPs in the interval chr7:23550265–29472427 (r^2^> 0.5) with *P*‐values lower than the threshold 1e‐8 remained. For further quality control, SNPs with a low frequency (–maf 0.05), high missing rate (–geno 0.05), and individuals with a high missing rate (–mind 0.06) were removed by PLINK, leaving a subset of 91 samples (41 for WT, 50 for OA) with 225 SNPs. Haplotypes were inferred using PHASE (ver 2.1) [72] with default settings. The population‐wide count of each haplotype was calculated according to the method published by Thomas et al. The probability of each haplotype pair used for calculation was based on the out_pairs file output by PHASE (ver 2.1) [73]. Considering the high diversity of the WT, which exhibited non‐identical haplotypes among all samples, haplotypes with a marginal count of ≥ 0.7 were plotted in the WT and OA abalone (Figure 2C). Except for Haplotype14 in the OA population, with a marginal count of 0.1, because it shows evidence for recombination between markers at 72 (26797726 bp) and 73 (26797737 bp).

### 5.4 Genotype Verification of Candidate SNP Variants

We genotyped the risk SNP rs26532113 variation between 23 orange‐muscle and 40 common abalone individuals using real‐time PCR (RT‐PCR) and Sanger sequencing. To isolate DNA, approximately 100 mg of muscle per abalone was shredded, DNA was extracted using phenol chloroform extraction methods, and the samples were placed in a refrigerator at –80°C. RT‐PCR was performed using abalone DNA and the following primers: 5′‐TATCACAGCAGACCCGTGAA‐3′ and 5′‐CCACTAGTGACCGGGTTGTT‐3′. PCR was performed under the following conditions: predenaturation at 94°C for 5 min, 30 cycles of denaturation at 94°C for 30 s, followed by annealing at 60°C for 30 s, and extension at 72°C for 1 min, before finally extending for 10 min. The PCR products were identified by Sanger sequencing (BioSune Co. Ltd., Shanghai, China).

### 5.5 Identification and Expression Analysis of ITGA8 Isoforms

To identify alternative splicing isoforms of *ITGA8*, RNA was extracted from abalone muscle using TRIzol reagent (Invitrogen, Carlsbad, CA, USA) following the manufacturer's instructions. RNA was reverse transcribed with the PrimeScript RT reagent Kit (Takara, Beijing, China). We employed RT‐PCR to amplify the *ITGA8* mRNA sequence, enabling the identification of three distinct *ITGA8* isoforms. RT‐PCR was performed using the following primers that spanned the mature isoforms from exons 1 to 11: 5′‐GTTTGTGAGCGGAGTCGTTGTG‐3′ and 5′‐ATGTCGCCGAGTTTGGTGAGGG‐3′. PCR was performed under the following conditions: predenaturation at 94°C for 5 min, 30 cycles of denaturation at 94°C for 30 s, followed by annealing at 62°C for 30 s, and extension at 72°C for 2 min, before finally extending for 10 min. For variant amplifications, the sizes of the different amplicons were measured by capillary electrophoretic fragment analysis using the same primers (Sangon Biotech Co. Ltd., Shanghai, China), and the sequences were identified by Sanger sequencing (BioSune Co. Ltd., Shanghai, China). We employed quantitative PCR (qPCR) using primers that precisely targeted distinct splice junctions in each of the multiple *ITGA8* isoforms to assess their expression proportions in different colored abalone muscles. The muscle samples of five wild‐type abalone and five orange‐muscle individuals were collected and stored at ‐80°C for qPCR. The Premix Ex Taq PCR system (Takara, Osaka, Japan) and FastStart Universal SYBR Green Master (ROX) (Roche, Mannheim, Germany) were used for qPCR. Three replicates of each biological sample were performed. The overall *ITGA8* expression levels (ΔCt) of the common sequence relative to the abalone control gene *ICG1* between orange muscle and wild‐type abalone muscles were compared using Student's *t*‐test [74]. To compare the relative expression proportion of each *ITGA8* isoform, we calculated the ΔCt of each isoform in relation to the common sequence and normalized it to the sum of the three isoforms. The Student's *t*‐test was used for data analysis. SYBR Green qPCR was performed using the following primers: (i) *ITGA8* common sequence primer: 5′‐AGCTGTTCTTCGACCTGACCT‐3′ and 5′‐CCGTGTTCTTCTCCACCACG‐3′; and (ii) control gene *ICG1* primer: 5′‐CAGGCTGACCATGCCAAGAA‐3′ and 5′‐GTGCTCACCGTCAGTTCCGT‐3′. The list of primers and probes used for TaqMan qPCR is provided in Table S13.

### 5.6 Protein Structural Modeling

MEGA (ver 10.1.8) was used for protein sequence prediction and *ITGA8* isoforms alignment. The protein sequences of various *ITGA8* isoforms were added to SWISS MODEL for homology modeling. The PDB files of three isoforms were downloaded, and the protein tertiary structure was predicted using ChimeraX (ver 1.5).

### 5.7 ATAC‐seq and Chromatin Accessibility Profiling

The muscle samples of three wild‐type abalone and three orange‐muscle individuals were collected. The samples were frozen in liquid nitrogen and stored at ‐80°C for ATAC‐seq, which was performed as previously reported [75]. Briefly, nuclei was extracted from samples, and the nuclei pellet was resuspended in the Tn5 transposase reaction mix. The transposition reaction was incubated at 37°C for 30 min. Equimolar Adapter 1 and Adatper 2 were added after transposition, PCR was then performed to amplify the library. After the PCR reaction, libraries were purified with the AMPure beads, and library quality was assessed with Qubit. The clustering of the index‐coded samples was performed on a cBot Cluster Generation System using TruSeq PE Cluster Kit v3‐cBot‐HS (Illumina) according to the manufacturer's instructions. After cluster generation, the library preparations were sequenced on an Illumina platform. Nextera adaptor sequences were first trimmed from the reads using skewer (0.2.2) [76]. These reads were aligned to a reference genome using BWA, with standard parameters. These reads were then filtered for high quality (MAPQ ≥ 13), nonmitochondrial chromosome, and properly paired reads (longer than 18 nt). All peak calling was performed with macs2 using ‘macs2 callpeak –nomodel –keepdup all –call‐summits’. For simulations of peaks called per input read, aligned and de‐duplicated BAM files were used without any additional filtering.

### 5.8 Electrophoretic Mobility Shift Assay of Proteins Bound to rs26532113

The orange‐muscle and wild‐type abalone muscles, each weighing 2–5 g, were thoroughly homogenized using liquid nitrogen. The proteins were extracted and stored at −80°C for future use. EMSA was performed using the Axl‐EMSA kit (Axl‐biotech, Guangzhou, China). The 24‐bp double‐strand oligonucleotide probes of different *ITGA8* genotypes of rs26532113 were synthesized and labeled with 5′ biotin (Axl‐biotech, Guangzhou, China). The 5′‐biotinylated probes of different genotypes were as follows: G‐wt genotype: TAAAGGGATCGGGTGGTCAGGCTT; A‐mut genotype: TAAAGGGATCAGGTGGTCAGGCTT. The same fragment without a 5′ biotin label was used as a competitor. The protein was incubated in solutions containing different genotypes of the labeled probes and unlabeled competitors in accordance with the manufacturer's protocol. The protein‐DNA complexes were separated using 5% native polyacrylamide gels, electro‐transferred onto a nylon membrane, and then cross‐linked using a UV‐light crosslinker (SCIENTZ 03‐II, SCIENTZ, China) at a wavelength of 254 nm (120 mJ/cm^2^). The signals were visualized using chemiluminescence detection.

### 5.9 DNA Pull‐Down Assay and Mass Spectrometry Analysis

Biotinylated probes corresponding to different *ITGA8* genotypes at rs26532113 were synthesized to selectively capture the proteins that interact with the DNA sequences encompassing this variant. The 5′‐biotinylated probes of different genotypes were as follows: G‐wt genotype: TAAGAGACGGCTAAAGGGATCGGGTGGTCAGGCTTGCTGACTTGGTTGA; A‐mut genotype: TAAGAGACGGCTAAAGGGATCAGGTGGTCAGGCTTGCTGACTTGGTTGA. DNA pull‐down assays were performed using a DNA pull‐down kit (Axl‐biotech, Guangzhou, China). Briefly, corresponding quantities of biotin‐labeled targeted DNA probe magnetic beads of different genotypes and an equal amount of negative control (NC) probe magnetic beads were prepared. The 2–5 g orange‐muscle and common abalone muscles were homogenized on ice with a homogenizer 5–10 times to extract nuclear proteins. Nucleic acid removal and pre‐washing of the prepared protein samples were performed. The pre‐washed protein samples were incubated with the corresponding probe‐magnetic bead complex for incubation. The magnetic beads were washed for times, and pulled‐down proteins were collected. The protein samples were stored at ‐80°C. Next, the protein samples were taken for PAGE‐silver staining detection. The protein samples were identified by Q Exactive hybrid quadrupole‐Orbitrap mass spectrometer (Thermo Fisher Scientific, San Jose, CA, USA). Protein identification was performed using MASCOT (http://www.matrixscience.com/) software by searching for Uniprot_*Aedis Aegypti*. Kyoto Encyclopedia of Genes and Genomes (KEGG) pathway analysis was performed on the differential proteins to help select a candidate protein.

### 5.10 Electrophoretic Mobility Shift Assay of ILF2 Binding to rs26532113

To evaluate whether two different *ITGA8* genotypes of rs26532113 can bind to abalone ILF2 protein, a pET‐28a (+) plasmid (Thermo Fisher Scientific, MA, USA) containing the fusion sequence of His‐tagged abalone *ILF2* was constructed. The 100 µL of BL21 (DE3) competent cells (Axl‐biotech, Guangzhou, China) were transfected with 1 µL of the constructed plasmid and subsequently cultured and induced. The protein was purified using a Ni‐NTA 6FF His‐Tag protein purification kit (Sangon Biotech, Shanghai, China), according to the manufacturer's instructions. Finally, the purified protein concentration was determined using the Nano‐800 spectrometer (Jiapeng Technology, Shanghai, China). An EMSA experiment was conducted using purified ILF2‐HIS protein. The probes of the two *ITGA8* genotypes of rs26532113 and the experimental procedure followed those described in the electrophoretic mobility shift assay of proteins bound to the rs26532113 part.

### 5.11 SNP‐Reporter Assay

To directly assess whether SNP rs26532113 variation in intron 3 can alter *ITGA8*‐mRNA alternative splicing, two minigenes were separately constructed in the pSPL3 vector (Waltham, Massachusetts, USA), including part of the gDNA sequences from exon 3 to exon 6 of abalone with wild‐type and SNP variations. Synthetic DNA fragments (General biol, Anhui, China) containing abalone *ITGA8* exons 3, 4, 5‐1, 5‐2, and 6 and the shortened corresponding introns (preserving wild‐type and variant SNP) were assembled into the intron of the pSPL3 vector. The libraries of synthetic fragments were cloned into reporters via two digestion XhoI (CTCGAG)‐BamHI (GGATCC) sites in the intron of pSPL3, and the recombinant plasmids were transfected into HEK293T cells for culture. Total RNA was extracted and reverse‐transcribed into cDNA after 24 h of transfection, and RT‐PCR was started using the following vector‐specific oligonucleotide primers: 5′‐TCTGAGTCACCTGGACAACC‐3′; 5′‐ATCTCAGTGGTATTTGTGAGC‐3′. The PCR products were evaluated using 1% agarose gel electrophoresis and further validated using Sanger sequencing (BioSune Co. Ltd., Shanghai, China).

### 5.12 siRNA‐Mediated Mouse‐ITGA8 Knockdown in RAW264.7 Cells

For knockdown of the mouse‐*ITGA8* gene, three siRNA were synthesized by Sangon Biotech Co. Ltd., Shanghai, China. The list of siRNA sequences can be found in Table S14. The siRNA was chosen for the experiment due to its highest knockdown efficiency and was synthesized from the following sequence: 5′‐GCUACUCACUGGACUUCUATT‐3′ and 5′‐UAGAAGUCCAGUGAGUAGCTT‐3′. The knockdown efficiency of mouse‐*ITGA8* was evaluated using qPCR with the following primers: *ITGA8*‐primer: 5′‐ACTGGAGAACTCTGAAACCTAATC‐3′ and 5′‐TTCCAGGTCCTCCCACTATAA‐3′; control gene GAPDH‐primer: 5′‐TGTGTCCGTCGTGGATCTGA‐3′ and 5′‐TTGCTGTTGAAGTCGCAGGAG‐3′. A negative control was used as a control. 2 × 10^6^ RAW264.7 cells were maintained at 70%–80% confluency and transfected with 100 nm siRNA using the Hieff Trans (Yeasen Biotechnology, Shanghai, China) according to the manufacturer's protocols. Experiments were performed 24 h after transfection and consisted of three replicates. To calculate the cell adhesion rate, 1.0 × 10^6^ cells were seeded onto 24‐mm round glass coverslips. After 24 h of transfection, the glass coverslip was soaked, incubated with EDTA solution (0.2 kg/L) for 10 min at 37°C, then washed with phosphate buffered saline (PBS) three times. The cells that were still adhered to the cover glass were photographed and counted. Parallel tests were performed three times per replicate. The adhesion rate was calculated as = (adhesion cell numbers/plate cell numbers) × 100%. For the oil red O staining test, 8.0 × 10^5^ cells were seeded onto 24‐mm round glass coverslips. After 24 h of transfection, the glass coverslip was rinsed twice with PBS, fixed with 4% paraformaldehyde for 10–15 mins, then stained with saturated oil red O solution for 8–10 min. After differentiation, soaking, and sealing, a glass cover slip was observed and photographed using a microscope. The lipid droplets were orange‐red to bright red, and the nuclei were light blue. The Aipathwell (ver 2.0) (Servicebio Technology, Wuhan, China) was used to analyze the distribution of oil droplets, and parallel tests were performed three times per repeat. The oil red positive area% was calculated as = (positive area/tissue area) × 100%. For cell images, 8 × 10^5^ cells were seeded onto 24‐mm round glass coverslips. After 24 h of transfection, the cells were fixed and imaged using a Hitachi SU8100 scanning electron microscope (Hitachi, Tokyo, Japan). To evaluate the absorption capacity of zeaxanthin, 8 × 10^5^ cells were seeded onto 24‐mm round glass coverslips. After 24 h of transfection, purified zeaxanthin (2 mL, Sigma, Buchs, Switzerland) with a media concentration of 4 µg/mL was added, and the cells were cultured for 16 h. Then, after removing the media, the cells on the plate were washed three times with PBS before harvesting nearly the same weight of cells between treatments by scraping in PBS (0.5 mL). The total zeaxanthin value of three replicates for each treatment group was determined using normal‐phase high‐performance liquid chromatography, following the method previously described for abalone tissues.

### 5.13 Overexpression of Different Mouse ITGA8 Isoforms in RAW264.7 Cells

To achieve overexpression of mouse *ITGA8*‐isoform 1 and its mutant (mouse *ITGA8*‐isoform 1‐Mut), corresponding overexpression plasmids were constructed using the pcDNA3.1 vector (Promega, WI, USA), with a custom empty vector plasmid serving as the negative control. 1.5 × 10^5^ RAW264.7 cells were maintained at 70%–80% confluency and transfected with 2 µg plasmid using Hieff Trans (Yeasen Biotechnology, Shanghai, China) according to the manufacturer's protocols. Experiments were performed 24 h after transfection and consisted of nine replicates. After 24 h of transfection, we verified the overexpression efficiency of mouse *ITGA8*‐isoform 1 and its mutant using qPCR with the following primers: *ITGA8*‐isoform 1‐primer: 5′‐GGCAGCTACTTCGGCTACTC‐3′ and 5′‐AGGGCCAGGGACAGTAGTAG‐3′; *ITGA8*‐isoform 1‐Mut‐primer: 5′‐CGTGTCTGGCGTTCAACTTG‐3′ and 5′‐TCTCACTGTGGCTCCAAACC‐3′; and control gene GAPDH‐primer: 5′‐TGTGTCCGTCGTGGATCTGA‐3′ and 5′‐TTGCTGTTGAAGTCGCAGGAG‐3′. To calculate the cell adhesion rate, 5.0 × 10^5^ cells were seeded onto 24‐mm round glass coverslips. Oil Red O staining was used to compare the changes in lipid accumulation capacity among different groups. After 24 h of transfection, 12 µL of purified zeaxanthin (Sigma, Buchs, Switzerland) at a media concentration of 1 mg/mL was added, and the cells were cultured for 16 h. The absorption capacities of zeaxanthin were compared among different groups. The specific methods for all detection indices were consistent with those used in the mouse *ITGA8*‐knockdown experiment.

### 5.14 Transcriptome Sequencing Analysis

The muscles of orange‐muscle and common abalone were collected, and three biological replicates were set in each group, with each replicate consisting of a mixture of three samples prepared for RNA extraction. Total RNA was used as the input material for the RNA‐seq library preparations and was sequenced on an Illumina Novaseq platform. Clean data were obtained by removing reads containing adapters, reads containing ploy‐N and low‐quality reads from the raw data. The paired‐end clean reads were aligned to the reference genome using Hisat2 (ver 2.0.5) [77]. The mapped reads of each sample were assembled using StringTie (ver 1.3.3b) [78] in a reference‐based approach. The featureCounts (ver 1.5.0‐p3) was used to obtain the read counts of each gene, and the fragments per kilobase million (FPKM) of each gene were calculated. Differential expression analysis was performed using the DESeq2 R package [79]. The resulting *P*‐values were adjusted using Benjamini and Hochberg's approach to control the false discovery rate. Genes with an adjusted *P*‐value ≤ 0.05 were classified as differentially expressed genes (DEGs). Gene Ontology (GO) and Kyoto Encyclopedia of Genes and Genomes (KEGG) pathway enrichment analysis of DEGs was implemented using the clusterProfiler R package, with a corrected *P*‐value < 0.05 considered significant.

### 5.15 4D‐Label Free Quantitative Phosphorylated Proteomic Analysis

The muscles of orange‐muscle and wild‐type abalone were collected, and five biological replicates were set in each group, frozen in liquid nitrogen, and stored at ‐80°C for phosphorylated proteomic analysis. SDT (4% SDS, 100 mm Tris‐HCl, 1 mm DTT, pH 7.6) buffer was used for sample lysis and protein extraction. The protein content was quantified using a BCA Protein Assay Kit (Bio‐Rad, USA). Protein digestion using trypsin was performed according to the filter‐aided sample preparation (FASP) procedure described by Matthias Mann. Phosphopeptide enrichment was performed using the High‐SelectTM Fe‐NTA Phosphopeptides Enrichment Kit according to the manufacturer's instructions (Thermo Fisher Scientific, MA, USA). After lyophilization, the phosphopeptide peptides were resuspended in 20 µL loading buffer (0.1% formic acid). LC‐MS/MS analysis was performed on a timsTOF Pro mass spectrometer (Bruker) coupled to a Nanoelute (Bruker Daltonics) for 60 min. The peptides were loaded on a C18‐reversed phase analytical column (homemade, 25 cm long, 75 µm inner diameter, 1.9 µm, C18) in buffer A (0.1% Formic acid) and separated with a linear gradient of buffer B (84% acetonitrile and 0.1% Formic acid) at a flow rate of 300 nL/min. The mass spectrometer was operated in the positive ion mode. The mass spectrometer collected the ion mobility MS spectra over a mass range of m/z 100–1700 and 1/k0 of 0.6–1.6, before performing ten cycles of PASEF MS/MS with a target intensity of 1.5k and a threshold of 2500. Active exclusion was enabled with a release time of 0.4 min. The MS raw data for each sample were combined and searched using MaxQuant for identification and quantitation analysis. Cluster 3.0 (http://bonsai.hgc.jp/~mdehoon/software/cluster/software.htm) and Java Treeview software (http://jtreeview.sourceforge.net) were used for hierarchical clustering analysis. The protein sequences of the selected differentially expressed proteins were locally searched using the NCBI BLAST+ client software (ncbi‐blast‐2.2.28 + ‐win32.exe) and InterProScan to find homologue sequences, then gene ontology (GO) terms were mapped, and sequences were annotated using the software program Blast2GO. Following annotation steps, the studied proteins were blasted against the online Kyoto Encyclopedia of Genes and Genomes (KEGG) database (http://geneontology.org/) to retrieve their KEGG orthology identifications and were subsequently mapped to pathways in KEGG. Enrichment analysis was applied based on the Fisher’ exact test, considering the whole quantified proteins as the background dataset. Benjamini–Hochberg correction for multiple testing was further applied to adjust derived *P*‐values. And only functional categories and pathways with *P*‐values under a threshold of 0.05 were considered significant.

### 5.16 Transmission Electron Microscopy

The fresh muscles of three orange‐muscle and common abalone were cut into 1 mm^3^ small tissue blocks using a scalpel in a Petri dish containing electron microscope fixating solution to minimize mechanical damage, such as pulling, contusion, and extrusion. The muscle blocks were quickly immersed in a new electron microscope fixating solution at 4°C for 2–4 h, and were rinsed three times for 15 min each time with phosphate buffer PB (0.1 m, pH 7.4). The samples were fixed with 1% osmic acid prepared with phosphate buffer PB (0.1 m, pH 7.4) at room temperature for 2 h away from light, before rinsing three times for 15 min each time with phosphate buffer PB (0.1 m, pH 7.4). The tissues were dehydrated with 30%–50%–70%–80%–95%–100%–100% alcohol for 20 min each time and 100% acetone twice for 15 min each time. The sample was permeated and embedded with acetone: 812 embedding agent = 1:1 for 2–4 h at 37°C. The embedded plate was polymerized in an oven at 60°C for 48 h, and the resin block was removed for use. An ultra‐thin microtome was used to cut the resin block into 60–80 nm ultra‐thin slices. The slices were stained under dark conditions, and dried overnight at room temperature. The images were analyzed using a Hitachi HT7800 transmission electron microscope (Hitachi, Tokyo, Japan).

### 5.17 Statistical Analysis

Data are shown as means ± SD values of the indicated number of independent experiments, unless otherwise noted in the figure legends. Figures are generated using GraphPad Prism (version 8.0). All statistical analyses were conducted with SPSS version 16.0. Student's *t*‐test was performed unless specified otherwise. ^*^ *P* < 0.05, ^**^ *P* < 0.01, ^***^ *P* < 0.001. Differences that did not reach statistical significance are indicated as ns (not significant).

## Author Contributions

The research was designed by X.H.W., W.Z.P., W.L., and C.H.K. X.H.W., W.Z.P., B.Y.Y., Y.W., B.L.P., and Y.G. performed the research. Z.K.H. and G.S.H. analyzed the data. New reagents were contributed by M.Q.H., L.W.N., and X.L. The illustrations were created by Y.Y.W., M.Z.H., G.J.L., and J.W.H. The team was supervised by C.H.K., W.W.Y., and W.L. The paper was written by X.H.W., W.Z.P., and Z.K.H.

## Funding

This work was supported by the National Key R&D Program of China (Grant 2018YFD0901401) to Caihuan Ke, the Seed Industry Innovation and Industrialization in Fujian Province (Grant 2021FJSCZY02) to Caihuan Ke, and Earmarked Fund for CARS (No. CARS‐49) to Weiwei You.

## Ethics Statement

The procedures and protocols used in this research were approved in accordance with the guidelines of the relevant institutional committees. Ethical approval was obtained from the Institutional Animal Care and Use Committee (IACUC) of Xiamen University (XMULAC20240335).

## Conflicts of Interest

The authors declare no conflicts of interest.

## Supporting information




**Supporting File 1**: advs74065‐sup‐0001‐SuppMat.docx.

**Supporting File 2**: advs74065‐sup‐0002‐Tables.xlsx.

## Data Availability

All data are available in the main text or the supplementary materials. The raw sequencing data generated in this study, including genome assembly, whole genome resequencing, RNA‐seq, and ATAC‐seq, have been deposited in the National Center for Biotechnology Information (NCBI) under the following BioProject accession numbers: PRJNA1146997, PRJNA1150372, PRJNA1153971, and PRJNA1153980, respectively. The *H.gigantea* genome assembly is available at Figshare (https://figshare.com/s/c7c8dbe6c4ef8a51e064). The mass spectrometry proteomics data have been deposited to the ProteomeXchange Consortium via the iProX partner repository with the dataset identifier PXD055772.
