# Supplementary material for: Genetic Control of Tissue Remodeling by a Non‐Coding SNP in ITGA8 Explains Carotenoid‐Based Color Polymorphism in Marine Mollusks
Source: Adv Sci (Weinh). 2026 Jan 28;13(27):e20527. doi: 10.1002/advs.202520527 (PMC13170236; doi:10.1002/advs.202520527)
Supplement: Supplementary file 1 — Supporting File 1: advs74065‐sup‐0001‐SuppMat.docx. [file ADVS-13-e20527-s002.docx]

Supporting Information

Genetic control of tissue remodeling by a non-coding SNP in ***ITGA8*** explains carotenoid-based color polymorphism in marine mollusks

Xiaohui Wei^1†^ , Wenzhu Peng ^1,6†^ , Zekun Huang^1,2,5†^ , Bingye Yang ^7†^ , Yiyu Wu^1^ , Minzhen Han^1^ , Yi Wang ^1^ , Bingling Peng^3^ , Guosheng Hu^3^ , Jiawei Hong^1^ , Guijia Liu^1^ , Linwei Nie^1^, Yang Gan ^1^ , Miaoqin Huang^1,2,4^ , Xuan Luo^4^ , Weiwei You^1,2,4*^, Wen Liu^3*^ and Caihuan Ke^1,2,4*^

^†^These authors contributed equally to this work

*Correspondence: [chke@xmu.edu.cn](mailto:chke@xmu.edu.cn)

**Figure S1
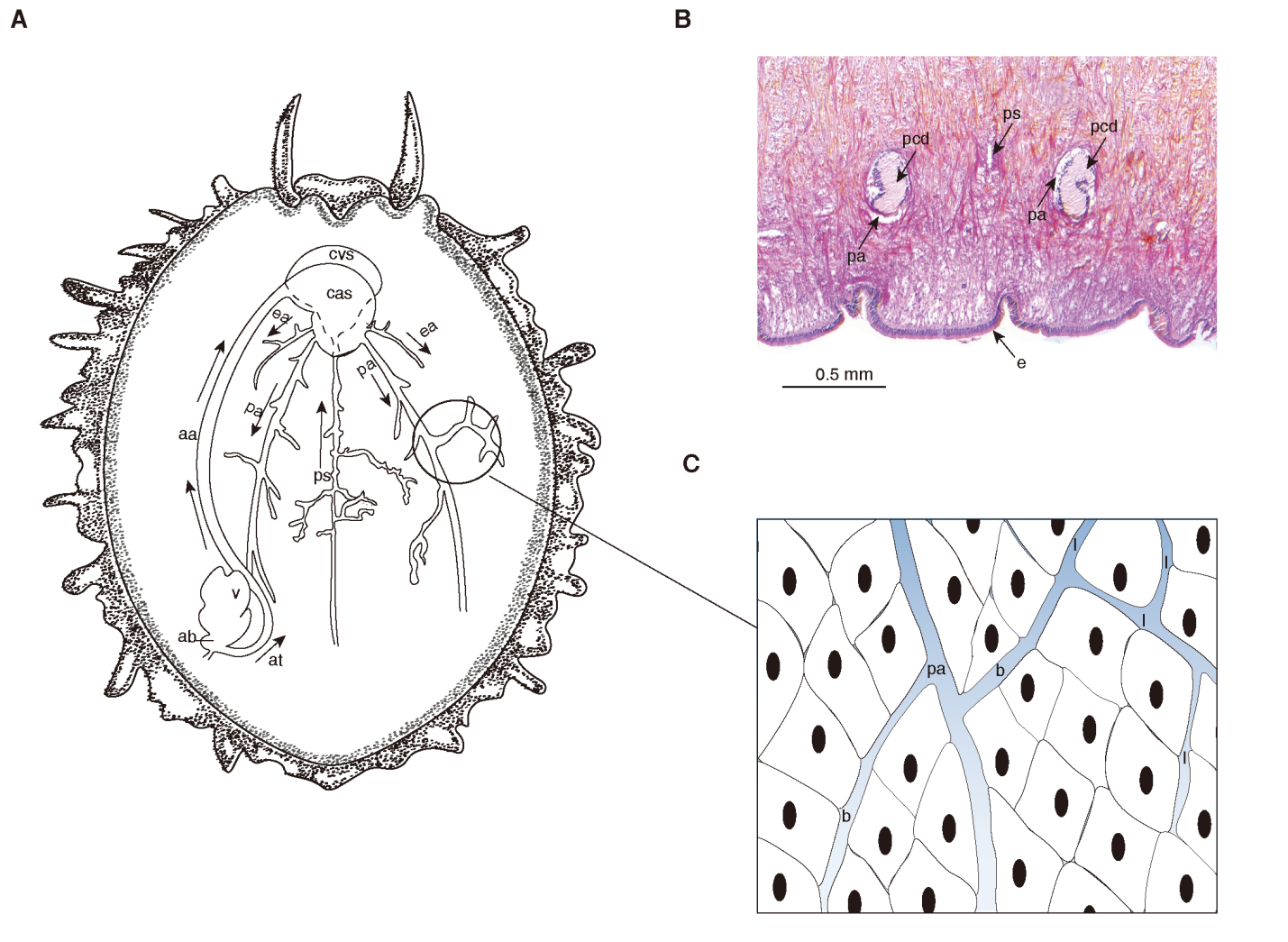
.** **Illustration of the cardiovascular system of abalone. A**, Diagram of the major elements of the cardiovascular system of abalone. cas: Cephalic arterial sinus; cvs: Cephalopedal sinus; pa: Pedal artery; ps: Posterior pedal sinus; ea: Epipodial artery; aa: Anterior aorta; ab: Aortic bulb; at: Common aortic trunk; v: Ventricle. **B**, Transverse wax section through the pedal mid-region of adult abalone. e: Pedal sole epithelium; pa: Pedal artery; ps: Posterior pedal sinus; pcd: Pedal ganglionated cords. **C**, Diagram of the microstructure of the cardiovascular system of abalone. pa: Pedal artery; b: Major arterial branch; l: Lacunar tissue spaces (LTS).

**Figure S2.** **Normal-phase HPLC chromatogram of the major carotenoid in tissues of different color abalone. A**, Peaks of the major carotenoids in muscle of orange-muscle abalone. **B**, Peaks of the major carotenoids in mantle of orange-muscle abalone. **C**, Peaks of the major carotenoids in muscle of wild-type abalone. **D**, Peaks of the major carotenoids in mantle of wild-type abalone. Peak 1 is β-carotene and peak 2 is zeaxanthin, and the monitoring wavelength of the chromatogram was 446 nm.

**
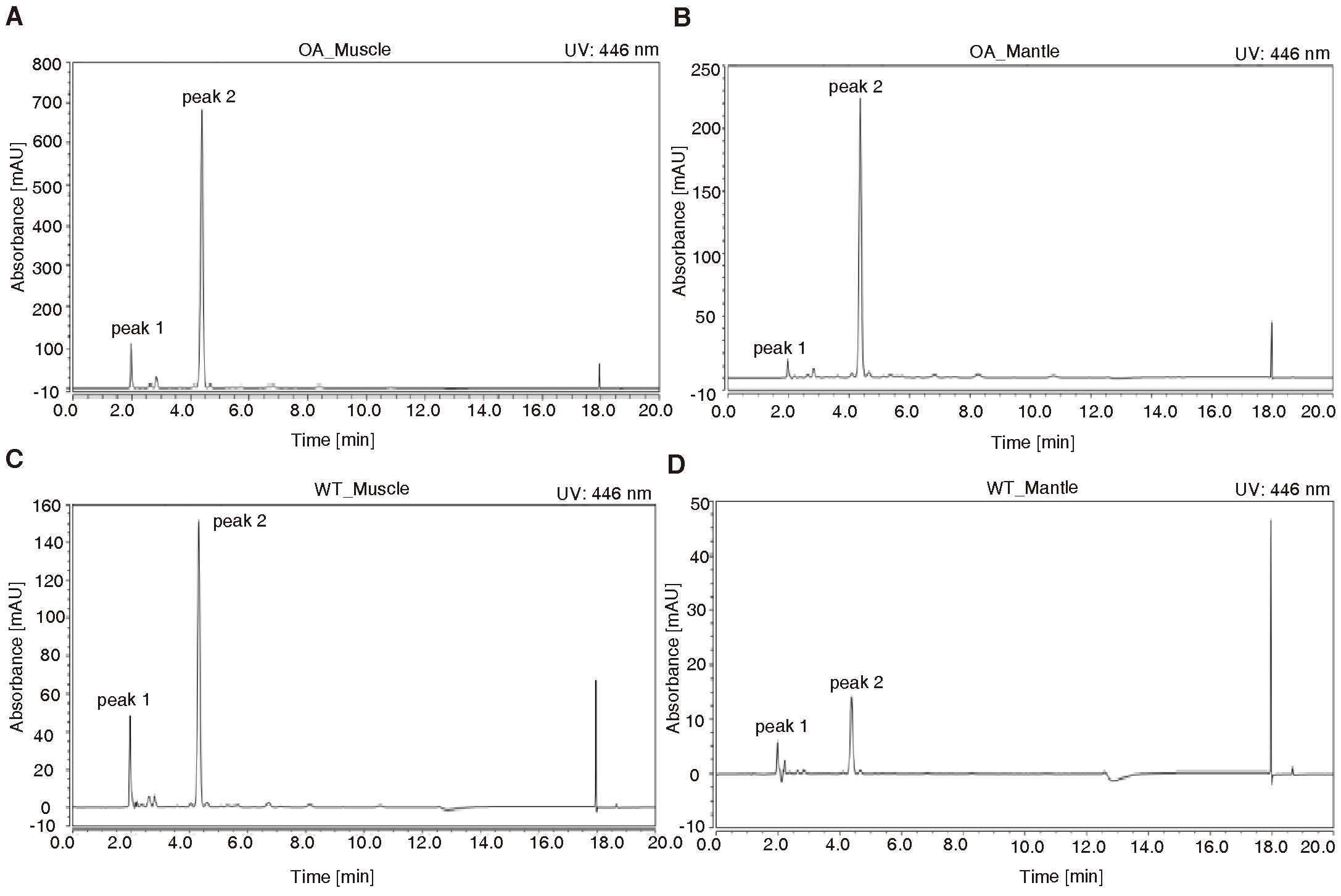
**

**
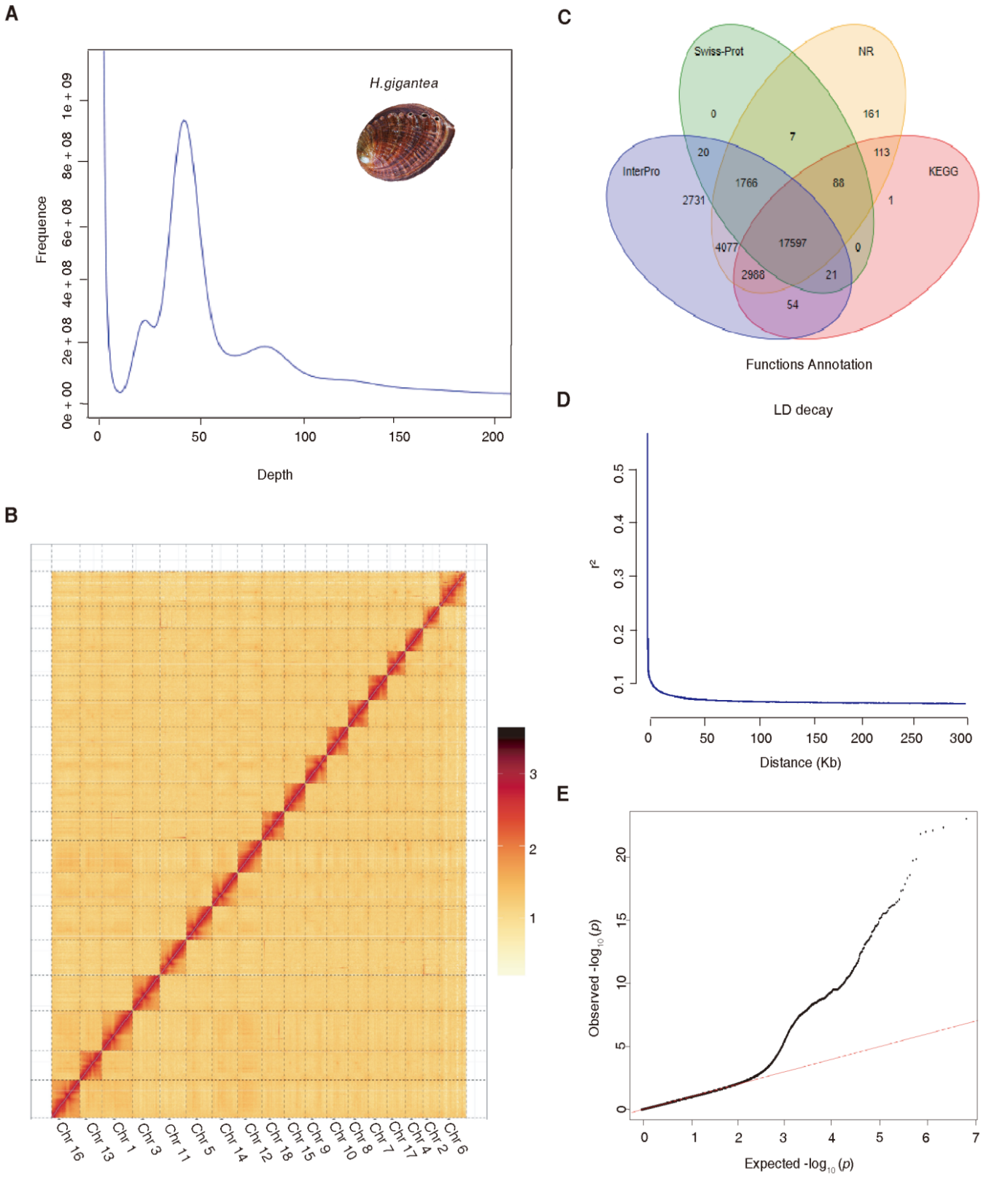
Figure S3.** **The genome assembly of *H. gigantea* and GWAS analysis of wild-type and orange-muscle abalone. A,** K-mer frequency distribution at K-mer = 17 depth. **B,** Hi-C chromosome contact map. Each block represents a Hi-C contact between two genomic loci within a 100-kb window. A darker colored block indicates a higher contact intensity. **C,** Venn diagram representing functional annotations from different databases, such as InterPro, Swiss-Prot, NR (Non-Redundant database), and KEGG (Kyoto Encyclopedia of Genes and Genomes). **D,** LD decay plots of orange-muscle and common abalone using r^2^ values. **E,** Q-Q plot for the *P*-values from GWAS analysis for wild-type and orange-muscle abalone.

**
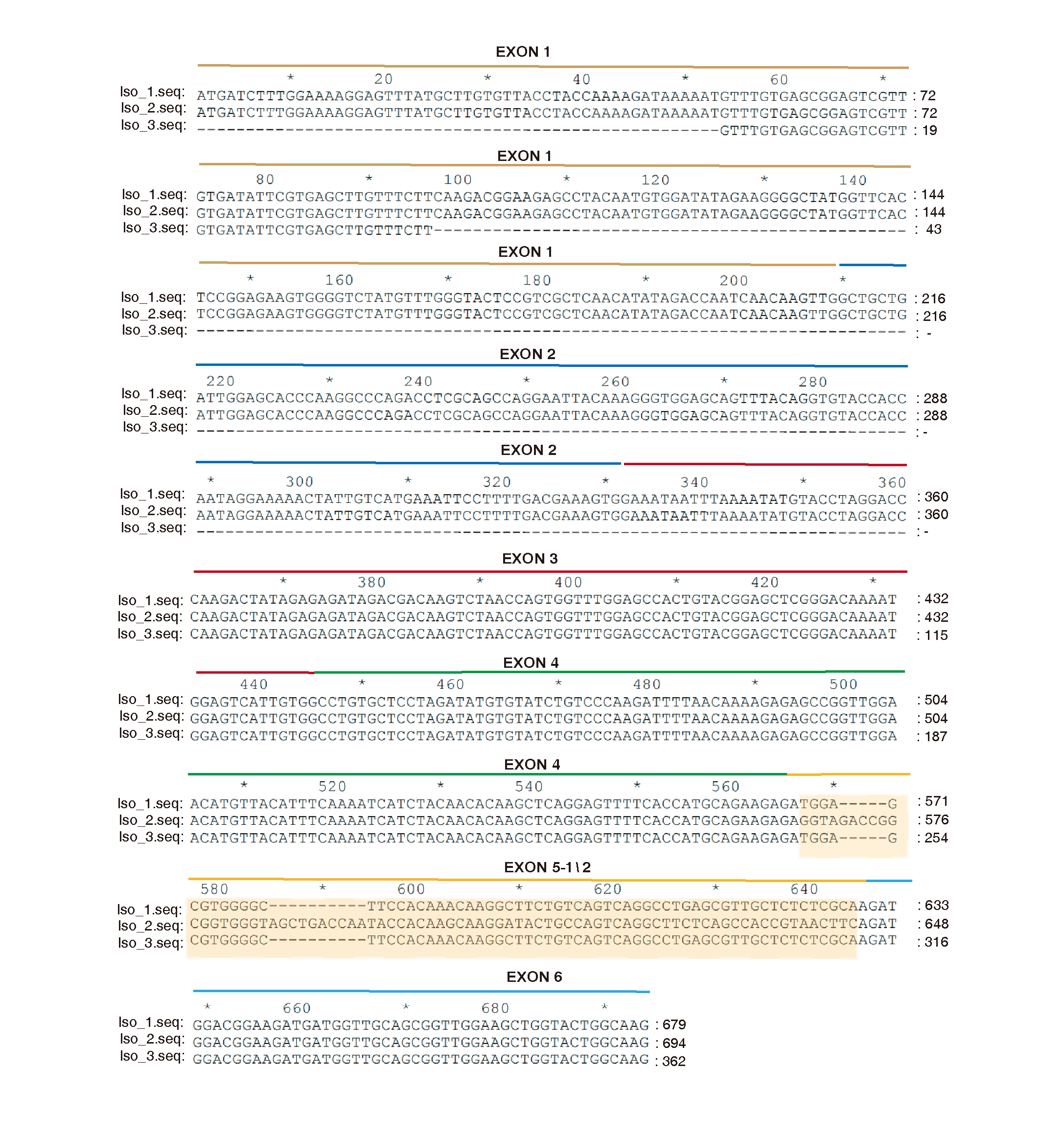
Figure S4. The alignment of cDNA sequences of the three *ITGA8* isoforms.** Alignment of exons 1-6 of the three *ITGA8* isoforms identified in abalone muscles. The light orange box indicates the different sequences in exon 5.

**
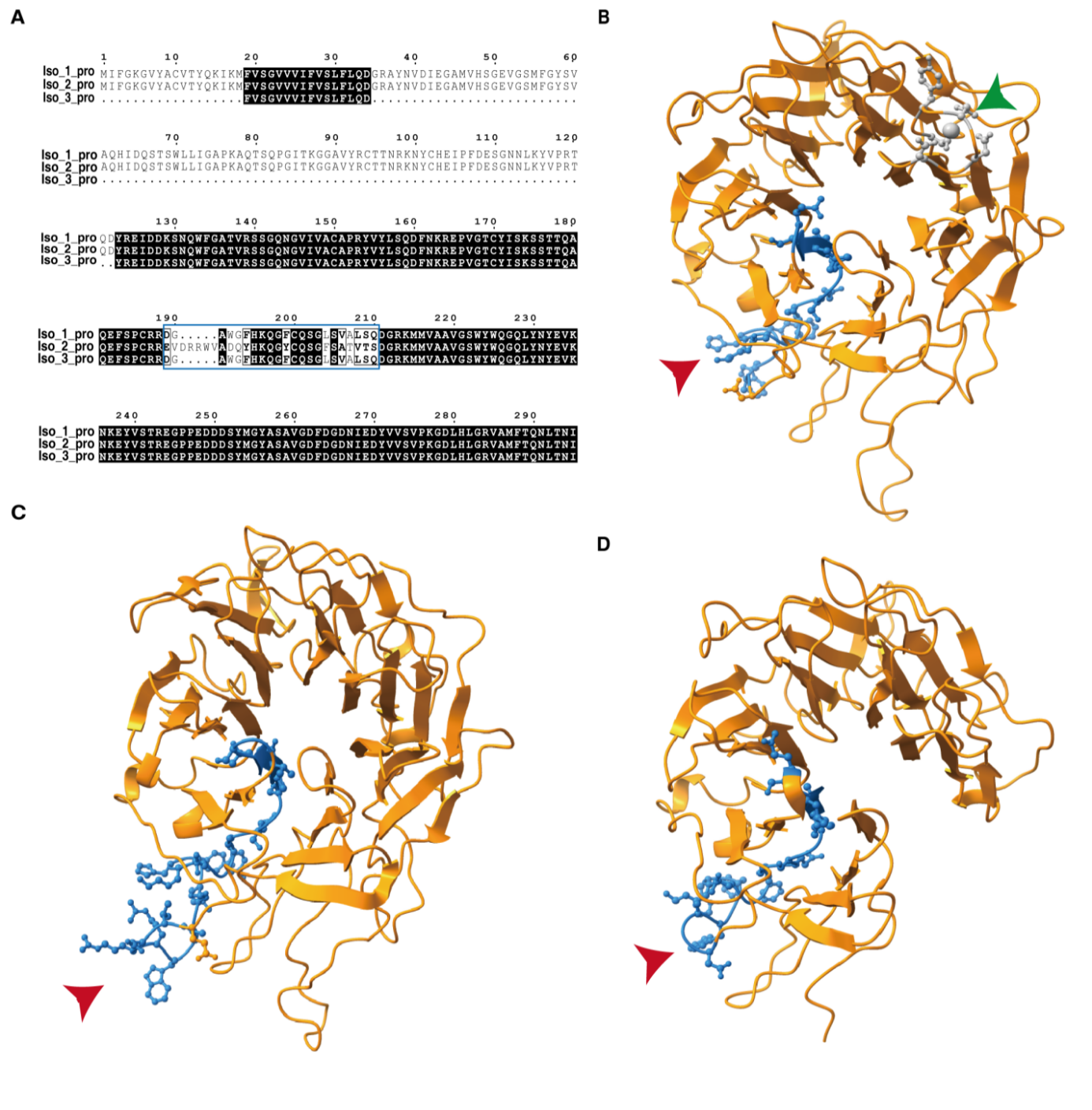
**

**Figure S5. Different amino acid sequences and tertiary structures of proteins encoded by three *ITGA8* isoforms in abalone. A,** The alignment of amino acid sequences encoded by three *ITGA8* isoforms; the blue box represents the amino acids encoded by different Exon 5. **B,** Diagram of the tertiary structure of the protein encoded by the *ITGA8*-isoform 1; the silver structure and green arrow represent the calcium ion binding site, and the blue structure and red arrow represent the variant sites. **C,** Diagram of the tertiary structure of the protein encoded by the *ITGA8*-isoform 2; the blue structure and red arrow represent the variant sites. **D**, Diagram of the tertiary structure of the protein encoded by the *ITGA8*-isoform 3; the blue structure and red arrows indicate sites identical to *ITGA8*-isoform 1, where variations occur in *ITGA8*-isoform 2.

**
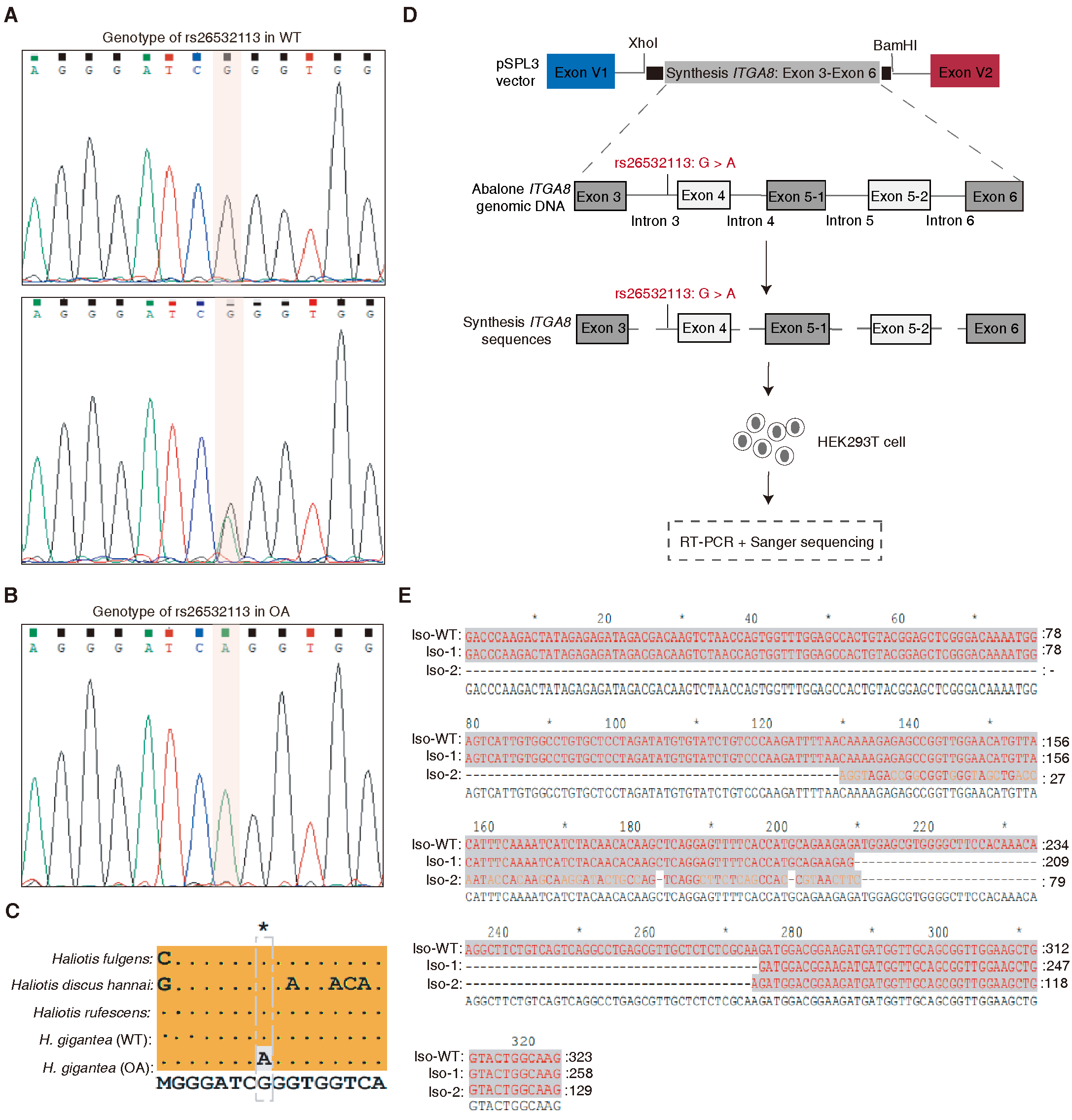
Figure S6. The functional verification of the risk SNP rs26532113. A** and **B,** The pink-highlighted peaks showed the genotypes of rs26532113 in the abalones with the different muscle colors obtained by Sanger sequencing. The genotype of rs26532113 is either homozygous GG (upper) or heterozygous GA (lower) in wild-type abalone (**A**), and the genotype of rs26532113 is homozygous AA in orange-muscle abalone (**B**). **C,** Abalone from various species aligned around the site of *ITGA8* rs26532113 variation. Silver curve highlights the location variation in orange-muscle abalone. **D,** Specific schematic diagram of the minigene assay for abalone *ITGA8* rs26532113. **E,** The sequences of three isoforms produced by minigene assays for abalone *ITGA8* rs26532113 variation by Sanger sequencing. Iso-WT: Isoform observed in the wild-type minigene; Iso-1 and Iso-2: Isoforms observed in the variant minigene.

**
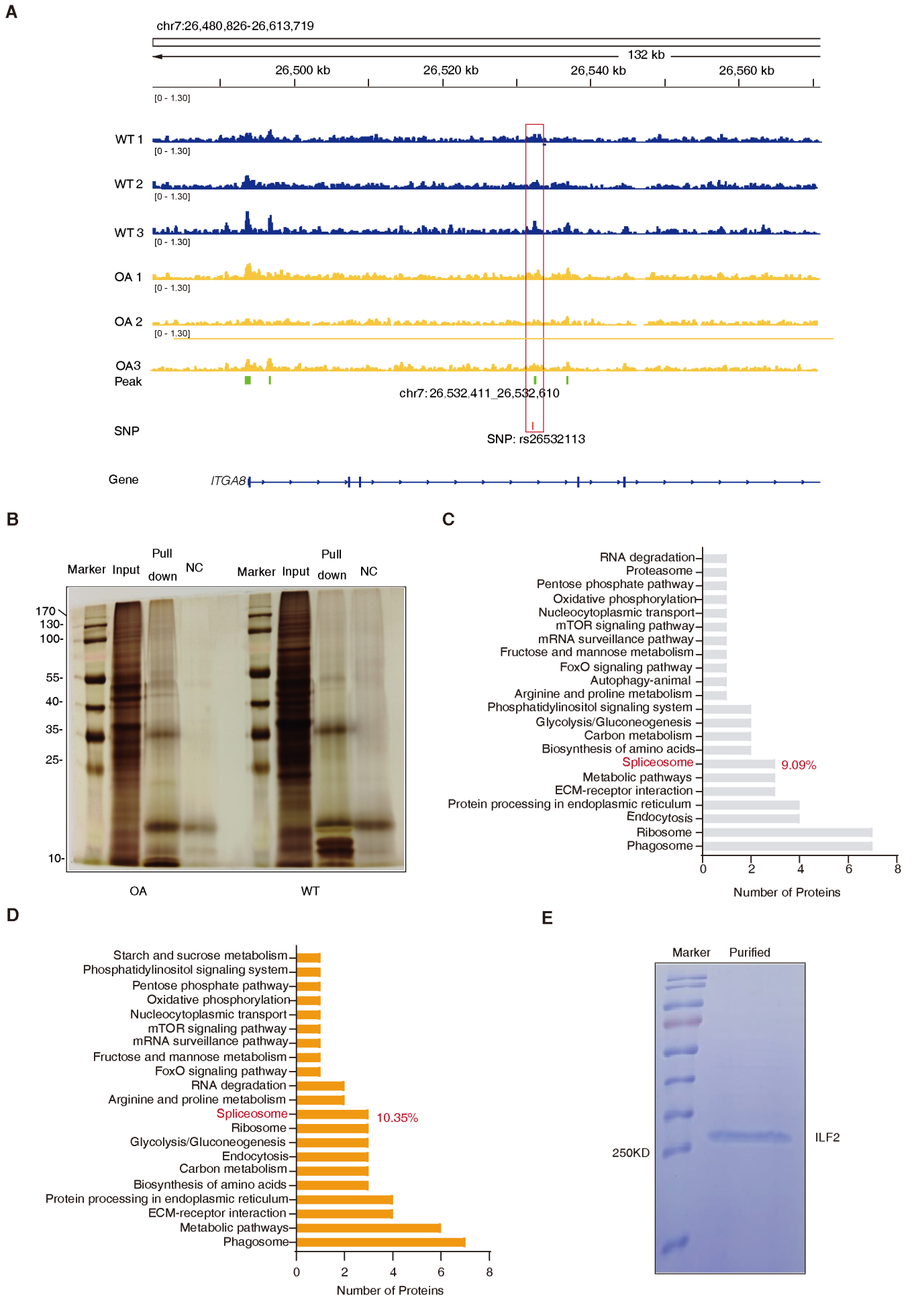
Figure S7. Mechanisms underlying alternative splicing regulated by the risk SNP rs26532113.** **A,** Chromatin accessibility tracks (ATAC-seq) close to the rs26532113 site of *ITGA8* in the wild-type and orange-muscle abalone sampled in this study. The green solid boxes represent ATAC-seq merged peaks; the red line represents the SNP site of *ITGA8*. **B,** Silver staining of proteins bound with different SNP genotypes by DNA pull-down assay. Distinct protein bands were observed using 49-bp probes including genomic sequences of wild-type and variant rs26532113. Input: All nuclear proteins extracted from each sample group; Pull down: Protein bands were obtained by probes each sample group; NC: Control. **C** and **D,** KEGG pathway enrichment analyses of protein complexes bound with the wild-type (**C**) and variant (**D**) SNP genotypes identified by mass spectrometry; The red-colored font and numbers represent the proportion of the spliceosome in the total protein numbers. **E,** Purified ILF2 protein.

**
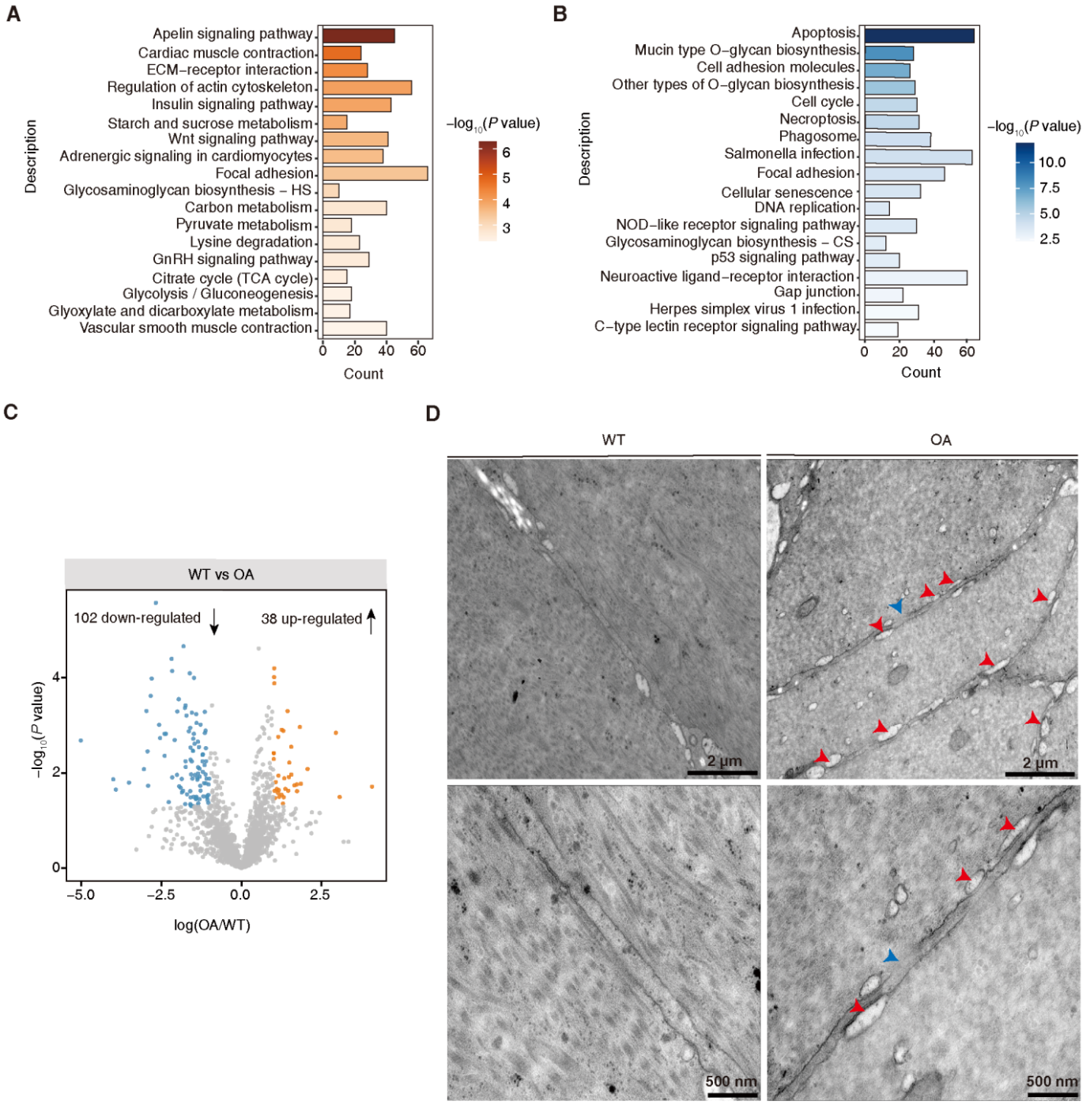
Figure S8. Multi****-omics analysis and cell architecture changes between muscles in wild-type and orange abalone. A,** The significantly enriched KEGG pathways for up-regulated genes in orange muscle. **B,** The significantly enriched KEGG pathways for down-regulated genes in orange muscle. **C,** The volcano map of the expression of the differentially expressed phosphopeptides in orange muscle compared to wild-type muscle. Phosphopeptides passing both thresholds (*P* value < 0.05, and FC >= 2.0 or FC <= 0.5) are colored. **D,** Representative TEM images showing changes in the tissue structures of wild-type and orange muscles (n = 3). Red arrow: Enhanced intercellular spaces; Blue arrow: Blurred cell membranes. Scale bars: 2 µm (top row) and 500 nm (bottom row).

**
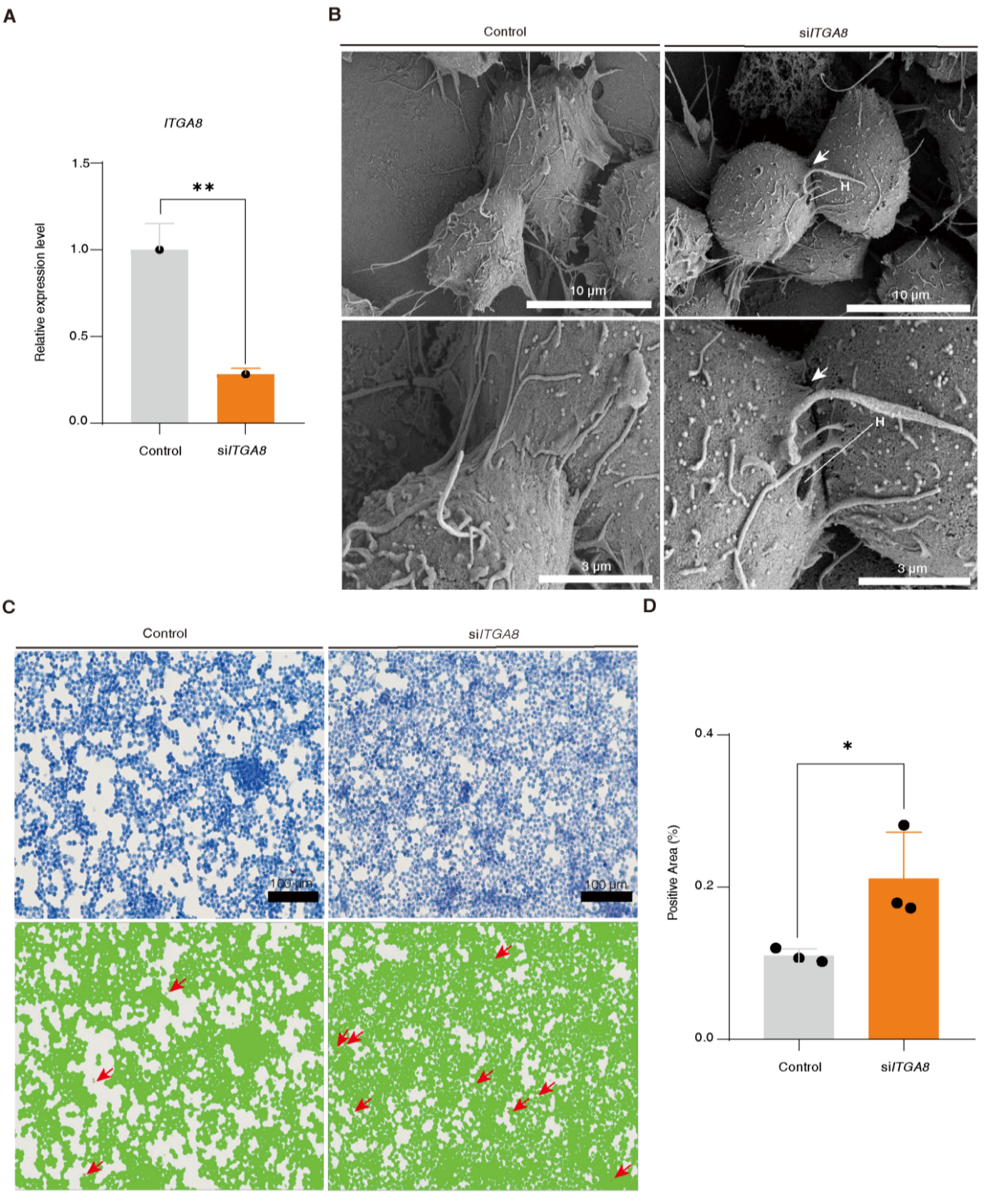
Figure S9. Functional verification of *ITGA8*-knockdown in cell structure and lipid in RAW264.7 cells. A,** The expressions of *ITGA8* were measured by RT-qPCR analysis between the control and *ITGA8*-knockdown groups. Statistical analyses: Student’s *t*-test. ***P* < 0.01. **B,** Representative scanning electron microscopy images showing changes in the cell structure of the control and *ITGA8*-knockdown groups (n = 3). H: Cellular holes; White arrow: Enlarged intercellular spaces. **C,** Representative images of Oil Red O staining between the control and *ITGA8*-knockdown groups (n = 3). Red arrow: Oil droplet. **D,** The Oil Red O staining-positive area was significantly increased in the *ITGA8*-knockdown group. Statistical analyses: Student’s *t*-test. **P* < 0.05.

**
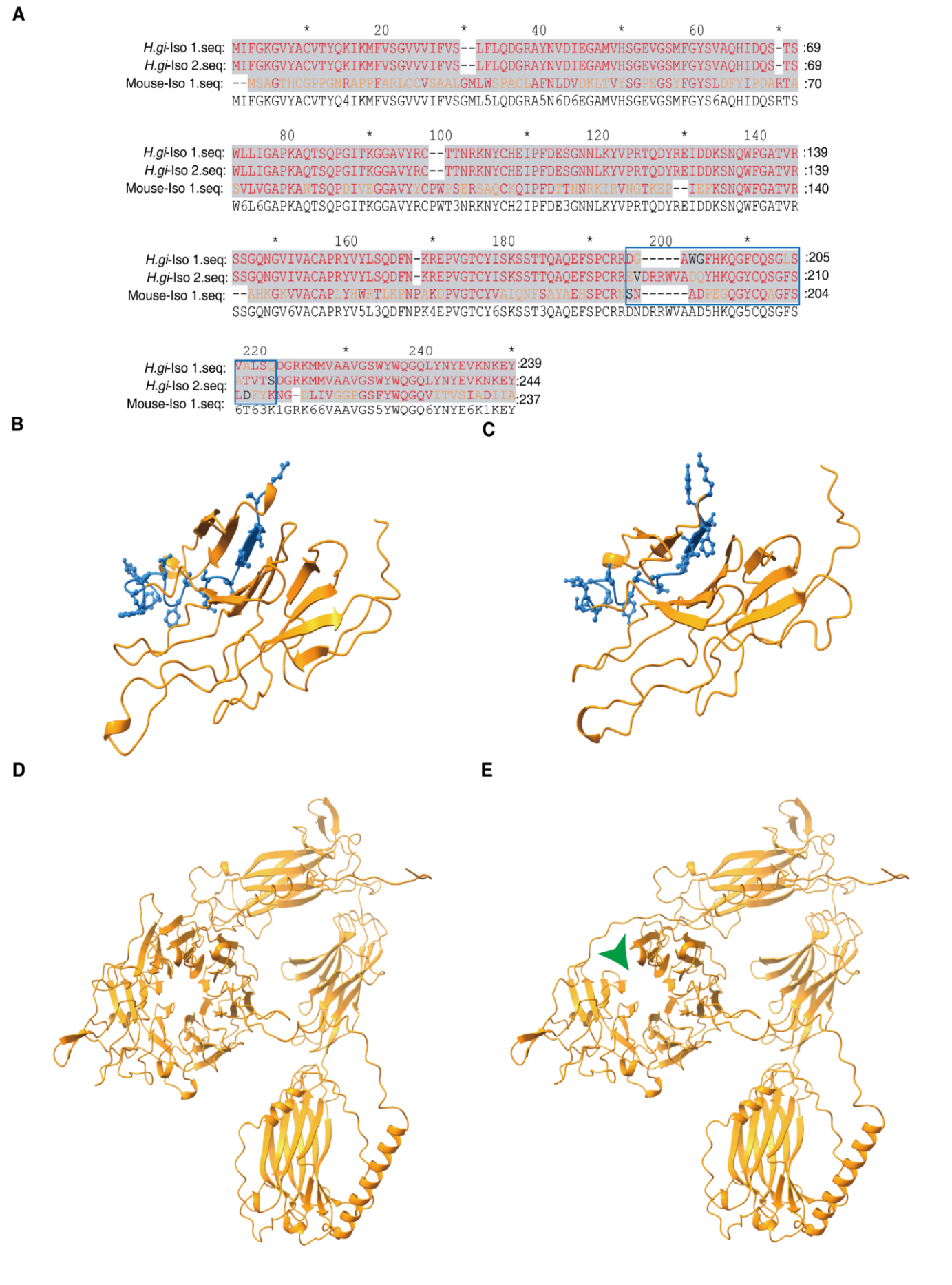
**

**Figure S10. Different amino acid sequences and tertiary structures of proteins encoded by different *ITGA8* isoforms in abalone and mouse. A,** Sequence alignment of the mouse *ITGA8-*isoform 1 and abalone *ITGA8*-isoform 1 and *ITGA8*-isoform 2. The blue box represents the different positions of the three sequences. **B,** Diagram of the tertiary structure of the protein encoded by the abalone *ITGA8-*isoform 1; the blue structure represents the variant sites. **C,** Diagram of the tertiary structure of the protein encoded by the mouse *ITGA8-*isoform 1; the blue structure represents the variant sites. **D,** Diagram of the tertiary structure of the full-length protein encoded by mouse *ITGA8*- isoform 1. **E,** Diagram of the tertiary structure of the full-length protein encoded by mouse *ITGA8*- isoform 1-Mut. The region indicated by the green arrows in the tertiary structure of the protein encoded by mouse *ITGA8*- isoform 1-Mut corresponds to the missing segment in the integrin β-propeller tertiary structure encoded by abalone *ITGA8*-isoform 3.


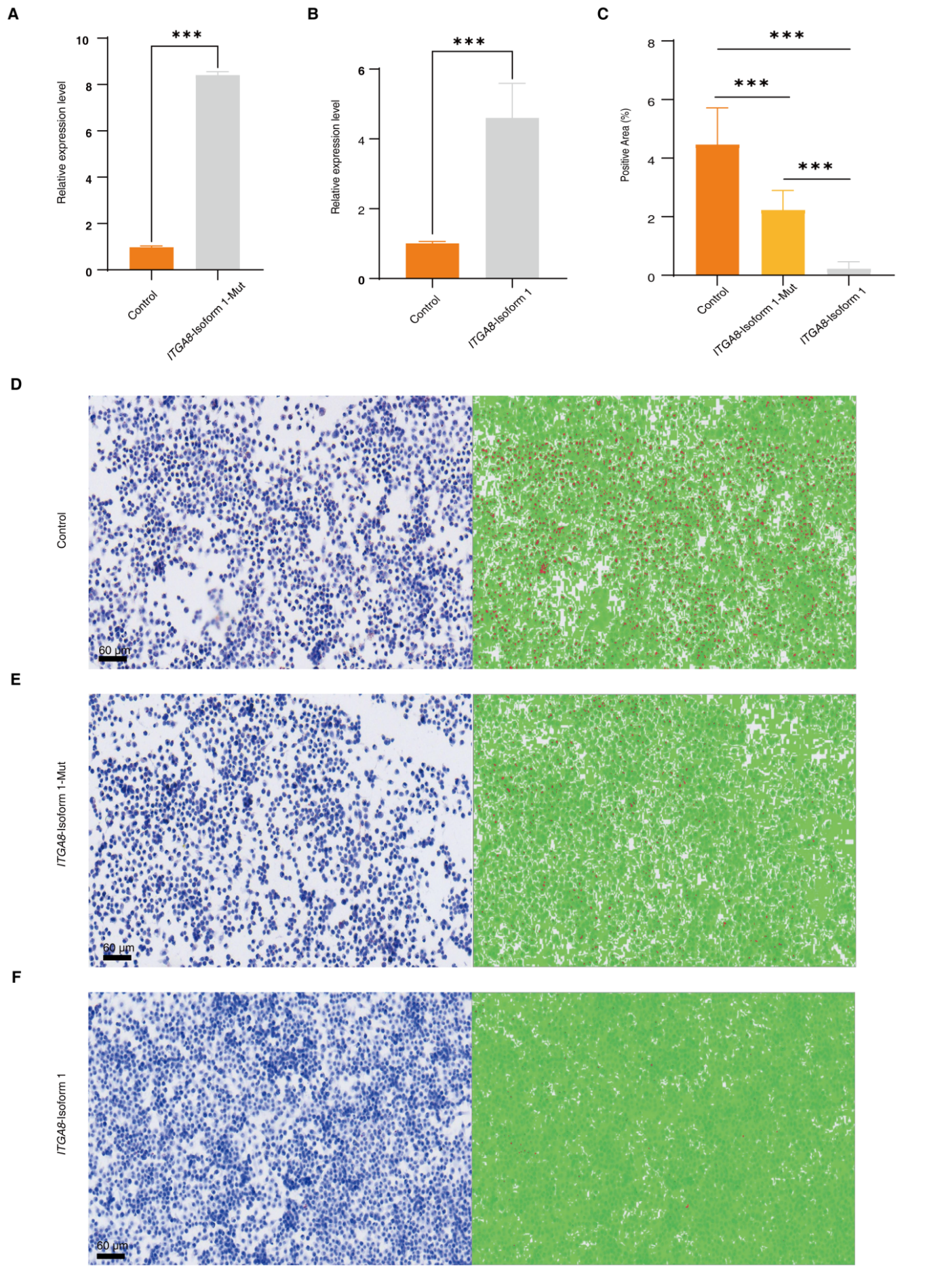


**Figure S11. Functional verification of *ITGA8* alternative splicing in RAW264.7 cells. A,** The expression of mouse *ITGA8*-isoform 1-Mut was measured by RT-qPCR analysis in control and overexpression groups. Statistical analyses: Student’s *t*-test. ****P* < 0.001. **B,** The expression of mouse *ITGA8*-isoform 1 was measured by RT-qPCR analysis in control and overexpression groups. Statistical analyses: Student’s *t*-test. ****P* < 0.001. **C,** The positive area of Oil Red O staining was compared among control and different overexpression groups. Statistical analyses: Student’s *t*-test. ****P* < 0.001. **D,**  Representative images of Oil Red O staining in the control group (n=9). **E,**  Representative images of Oil Red O staining in the mouse *ITGA8*-isoform 1-Mut overexpression group (n=9). **D,**  Representative images of Oil Red O staining in the mouse *ITGA8*-isoform 1 overexpression groups (n=9).
